# Plant immunity suppression by an exo-β-1,3-glucanase and an elongation factor 1α of the rice blast fungus

**DOI:** 10.1038/s41467-023-41175-z

**Published:** 2023-09-07

**Authors:** Hang Liu, Xunli Lu, Mengfei Li, Zhiqin Lun, Xia Yan, Changfa Yin, Guixin Yuan, Xingbin Wang, Ning Liu, Di Liu, Mian Wu, Ziluolong Luo, Yan Zhang, Vijai Bhadauria, Jun Yang, Nicholas J. Talbot, You-Liang Peng

**Affiliations:** 1 Ministry of Agriculture and Rural Affairs Key Laboratory for Crop Pest Monitoring and Green Control, China Agricultural University, Beijing, 100193 China; 2 The Sainsbury Laboratory, University of East Anglia, Norwich Research Park, Norwich, NR4 7UH UK

**Keywords:** Pathogens, Cell death and immune response, Fungal pathogenesis, Microbe

## Abstract

Fungal cell walls undergo continual remodeling that generates β-1,3-glucan fragments as products of endo-glycosyl hydrolases (GHs), which can be recognized as pathogen-associated molecular patterns (PAMPs) and trigger plant immune responses. How fungal pathogens suppress those responses is often poorly understood. Here, we study mechanisms underlying the suppression of β-1,3-glucan-triggered plant immunity by the blast fungus *Magnaporthe oryzae*. We show that an exo-β-1,3-glucanase of the GH17 family, named Ebg1, is important for fungal cell wall integrity and virulence of *M. oryzae*. Ebg1 can hydrolyze β-1,3-glucan and laminarin into glucose, thus suppressing β-1,3-glucan-triggered plant immunity. However, in addition, Ebg1 seems to act as a PAMP, independent of its hydrolase activity. This Ebg1-induced immunity appears to be dampened by the secretion of an elongation factor 1 alpha protein (EF1α), which interacts and co-localizes with Ebg1 in the apoplast. Future work is needed to understand the mechanisms behind Ebg1-induced immunity and its suppression by EF1α.

## Abstract (web summary)

Fungal cell walls release β-1,3-glucan fragments that trigger plant immunity. Here, the authors show that a glucanase (Ebg1) of the blast fungus *Magnaporthe oryzae* suppresses plant immunity by hydrolyzing β-1,3-glucan. At the same time, Ebg1 induces plant immune responses that are dampened by a fungal protein that interacts with Ebg1.

## Introduction

The cell wall is the outermost layer of fungal cells, composed of diverse polysaccharides interconnected into a three-dimensional structure to shape and protect fungal cells^1^. The fungal cell wall is also dynamic, undergoing remodeling to maintain its integrity, which is required for hyphal growth and development, infection-related morphogenesis and responses to environmental stress^2^. Cell wall remodeling involves fragmentation and re-linkage of polysaccharides by a series of glycosyl hydrolase (GH) family proteins. It has been reported that chitinases Cts1 and Cts2 of the GH18 family in *Ustilago maydis*^3^, the endo-β-1,3-glucanases ENG1 (GH81), ENG2-5 (GH16) and the β-1,3-glucanosyltransferase Gels (GH72) in *Aspergillus fumigatus*^4–7^, play a crucial role in cell wall remodeling. Since the cell wall is the primary contact point of pathogenic fungi with host cells, oligo- and poly-saccharides generated during the cell wall remodeling may leak into the apoplastic region of the fungus-host interface to trigger host immune responses. Oligomers of chitin^8–12^ and β-1,3-glucans^13–16^ are well-known pathogen-associated molecular patterns (PAMPs) and induce immune responses, both in plant and mammalian cells. For successful infection, fungal pathogens have evolved a battery of mechanisms to prevent elicitation of host defense responses by PAMPs. They may secrete effectors that bind to and prevent these oligosaccharide PAMPs from being recognized by host cells^17,18^. Fungal pathogens also secrete GHs and modifying enzymes, which degrade or modify polysaccharides into PAMP-inactive forms. For instance, *Verticillium dahliae* utilizes the polysaccharide deacetylase PDA1 to deacetylate chitin into chitosan^19^, and fungal pathogens harbor multiple chitinases (GH18) to digest chitin oligomers for evading plant recognition^3,20^. Meanwhile, many GH family proteins, including those GH enzymes that degrade plant cell wall polymers to provide nutrients for pathogen invasion^21,22^, may also induce plant immunity as PAMPs. One such GH is EIX, a GH11 protein in *Trichoderma viride* implicated in xylan degradation and the first characterized PAMP in plants^23,24^. However, it remains largely unknown how fungal pathogens mask these GH proteins to prevent them from causing plant immune responses.

*Magnaporthe oryzae* is the causative agent of rice blast disease, one of the most destructive crop diseases worldwide^25^. In the cell wall of *M. oryzae*, chitin and β-1,3-glucan are the two major forms of polysaccharide^2^. To suppress chitin-triggered immunity, the pathogen specifically expresses and secretes the chitin-binding effector Slp1^14,17^ and a chitinase MoChia1 (GH18) involved in hydrolyzing oligo-chitins into monomeric GlcNAc, during infection^26,27^. Furthermore, *M. oryzae* synthesizes and accumulates α-1,3-glucan on the surface of the cell wall to sequester β-1,3-glucan from plant recognition^28^. In addition, *M. oryzae* expresses several β-1,3-glucanosyltransferases (GH72) that are important for cell wall remodeling and virulence^29^. However, the *M. oryzae* genome encodes a much larger repertoire of GHs that have not been functionally characterized^30,31^, including seven GH17 family members similar to Scw4p, Scw10p, Scw11p, and Bgl2p, which act as endo-β-1,3-glucanases during cell wall remodeling in *Saccharomyces cerevisiae*^32–34^.

This study set out to investigate how *M. oryzae* safeguards cell wall remodeling involving β-1,3-glucan modification during infection. We identified a fungal-conserved exo-β-1,3-glucanase of the GH17 family, named Ebg1, which is vital for cell wall integrity and virulence of *M. oryzae*. Ebg1 degrades β-1,3-glucan into glucose to avoid β-1,3-glucan-triggered plant immunity. However, Ebg1 itself is also a PAMP. Interestingly, *M. oryzae* possesses a EF1α protein which interacts and co-localizes with Ebg1 in the apoplastic space to prevent it from triggering host immune responses.

## Results

### *Magnaporthe oryzae* gene *EBG1* is required for cell wall remodeling and pathogenicity

**Fig. 1:**
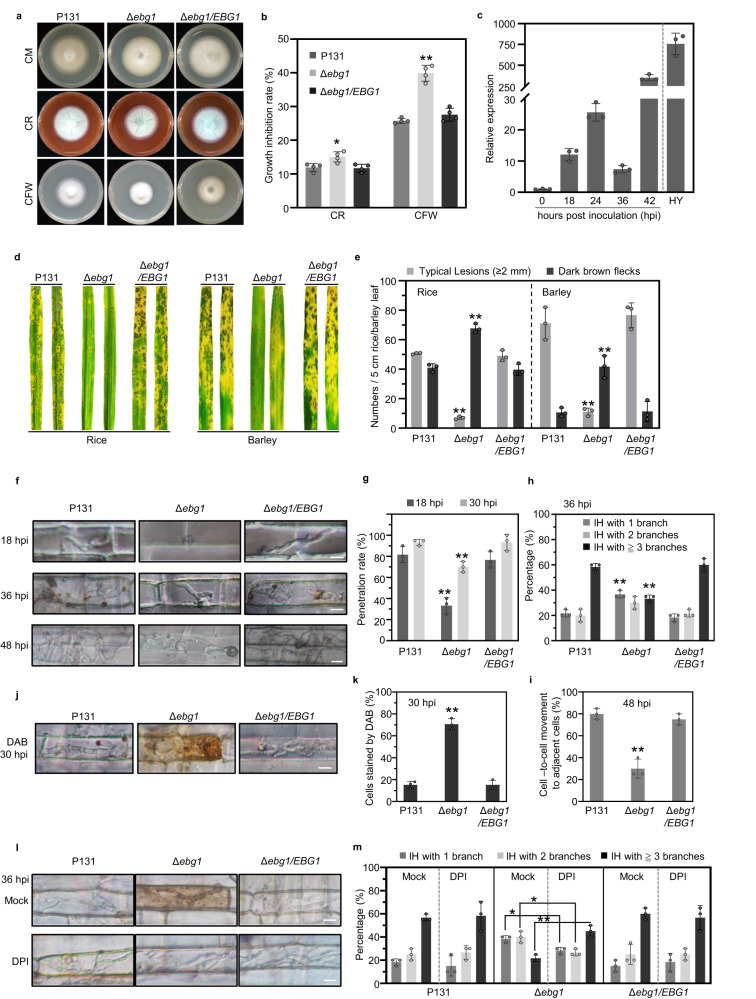
*Magnaporthe oryzae EBG1* is important for the cell wall integrity, virulence, invasive growth, and suppressing host ROS. **a**, **b** Δ*ebg1* mutants display defects in cell wall integrity. The wild-type P131, the *EBG1* deletion mutant Δ*ebg1* and a complemented transformant Δ*ebg1*/*EBG1* were cultured on the complete medium (CM) plates supplemented with 200 μg/ml CR or 100 μg/ml CFW at 28°C for 5 days (**a**), and their growth inhibition rates were calculated in (**b**). CR, Congo Red; CFW, Calcofluor white. **c** Relative expression levels of *EBG1* in vegetative hyphae (HY) and at different infection stages. RNAs were extracted from inoculated barley leaves at indicated hours post inoculation (hpi) and vegetative hyphae. Relative expression of *EBG1* referred to *MoActin* was calculated and the value at 0 hpi was set as 1. **d**, **e** Δ*ebg1* mutants show significantly reduced virulence on both rice and barley leaves. Detached leaves were sprayed with conidia suspensions (5 × 10^4^ spores/ml) of P131, Δ*ebg1,* and Δ*ebg1*/*EBG1* strains, and photographed at 5-day post inoculation (dpi) (**d**). Typical lesions and dark brown spots formed on rice and barley leaves were counted per leave (**e**). **f**–**i** Δ*ebg1* mutants are reduced in invasive growth. Rice sheath were inoculated with conidia suspensions of P131, Δ*ebg1*, and Δ*ebg1*/*EBG1* strains. The hyphal growth was observed and photographed at 18, 36, and 48 hpi with a Nikon 90i microscope (**f**). Scale bars = 20 μm. Δ*ebg1* mutants are reduced in appressorial penetration rates, which were calculated at 18 and 30 hpi (**g**). The percentage of distinct types of infection hyphae, e.g. one branch, two branches, and three or more branches, were examined at 36 hpi (**h**). IH, infection hyphae. The percentages of cell-to-cell movement of infection hyphae were calculated at 48 hpi (**i**). **j**, **k** Δ*ebg1* mutants induce ROS production in infected rice sheath cells. Rice sheath cells inoculated with the conidia suspensions (1 × 10^5^ spores/ml) of P131, Δ*ebg1*, and Δ*ebg1*/*EBG1* strains were stained with DAB at 30 hpi (**i**). Scale bars = 20 μm. The percentages of DAB-stained cells versus infected cells were calculated (**j**). **l**, **m** Diphenyleneiodonium (DPI), an inhibitor of NADPH oxidase, can partially restore the invasive growth of Δ*ebg1* mutant. Inoculated rice sheath cells were treated at 12 h post inoculation with 0.5 μM DPI dissolved in 1% DMSO and with 1% DMSO as a mock treatment. The growth of infection hyphae was observed and photographed at 36 hpi using a Nikon 90i microscope(**l**). Scale bars = 20 μm. Meanwhile, infection sites with distinct types of infection hyphae, e.g. one branch, two branches, and three or more branches, were scored to calculate their percentages (**m**). IH, infection hyphae. For all the above statistics, error bars denote standard deviations from three biological replicates. ** and * indicate *p* < 0.01 and *p* < 0.05 significant differences compared with the corresponding WT controls. One-way ANOVA with post-hoc Turkey tests were used in (**b**), (**c**), (**e**), (**g**–**i**) and (**k**), and two-tailed Student’s t-test were used in (**m**). Source data with statistic analysis are provided in a Source data file.

The *M. oryzae* genome contains seven GH17 genes encoding putative β-1,3-glucanases: *MGG_04582*, *MGG_00863*, *MGG_10591*, *MGG_09619*, *MGG_04689*, *MGG_06023*, and *MGG_10400*^30,31^ (Supplementary Fig. 1). To investigate their roles in cell wall remodeling and plant infection, we generated targeted gene deletion mutants for each of these genes, including *MGG_04582* named as *EBG1*, which shows similarity at its C-terminus to the *S*. *cerevisiae* Scw11p of GH17 involved in cell wall integrity^34^ (Supplementary Fig. 1). Bioassays showed that the two deletion mutants of *EBG1* (Supplementary Fig. 2a, b) are similar to the wild-type strain P131 in mycelial growth, conidiation, conidial germination and appressorium formation (Supplementary Fig. 2c–h). However, as shown in Fig. 1a, b, ∆*ebg1* mutants are highly sensitive to cell wall perturbing reagents such as calcofluor white (CFW) and congo red (CR). When grown on complete medium supplemented with CFW or CR, mycelial growth of P131 was reduced by 25.8% and 10.9%, respectively, whereas growth of ∆*ebg1* mutants was reduced by 41.5% and 14.3%, respectively. RT-qPCR analysis indicated that *MGG_04582* is constitutively expressed in vegetative hyphae, and exhibited differential expression during plant infection and invasive growth (Fig. 1c). When considered together, these observations indicate that *MGG_04582* is involved in cell wall remodeling during hyphal growth and plant infection.

We next assayed the effect of deletion of *EBG1* on fungal pathogenicity. Following spray inoculation of conidia on rice and barley leaves, the wild-type and ∆*ebg1/EBG1* complementation strains generated typical spreading blast lesions mixed with some smaller dark brown flecks, whereas the ∆*ebg1* mutant mainly formed tiny dark brown flecks mixed with very few typical spreading blast lesions (Fig. 1d, e), indicating that *EBG1* is important for virulence. In addition, the total number of lesions formed by the ∆*ebg*1 mutant was significantly less than those formed by the isogenic wild-type and complementation strains (Fig. 1d, e), suggesting that *EBG1* may also be involved in the initial infection of host cells.

### *EBG1* is important for invasive hyphae growth and suppression of host ROS during rice blast infection

To understand how *EBG1* is involved in virulence, we compared the infection process of the wild-type P131, the ∆*ebg1* mutant, and ∆*ebg1/EBG1* complementation strains in rice leaf sheaths and barley leaves. As shown in Fig. 1f–h, at 18-h post-inoculation (hpi), over 76% of appressoria formed by the wild-type and ∆*ebg1/EBG1* complementation strains penetrated rice leaf sheath cells and developed primary infection hyphae (IH), while only 33% of ∆*ebg1* mutant appressoria penetrated rice cells. At 30 hpi, the appressorium penetration rate of P131 and ∆*ebg1/EBG1* reached 93%, but only 70% for the ∆*ebg1* mutant. Furthermore, at 36 hpi, more than 58% of IH of P131 and ∆*ebg1/EBG1* formed multiple branches in primary invaded rice cells, whereas only 33% of IH of ∆*ebg1* formed multiple branches, indicating that development of ∆*ebg1* IH inside plant cells is attenuated. In addition, at 48 hpi, IH of P131 and ∆*ebg1/EBG1* at more than 75% sites expanded into adjacent rice cells, while only 30% of IH of ∆*ebg1* proliferated into adjacent cells (Fig. 1f, i). The infection process of the three strains in barley leaf cells was similar to that observed in rice leaf sheath cells (Supplementary Fig. 3a–c). Taken together, we conclude that *EBG1* is important for efficient appressorium-mediated penetration and invasive hyphal growth. We observed that ∆*ebg1* predominantly formed tiny dark brown flecks following plant infection (Fig. 1d, e) and we therefore, wondered whether *EBG1* plays a role in suppressing host immune responses, such as the generation of reactive oxygen species (ROS). We, therefore examined ROS accumulation in both rice leaf sheath cells and barley leaf epidermal cells infected by P131, ∆*ebg1*, and ∆*ebg1/EBG1* strains using 3,3′-diaminobenzidine (DAB), a dye that detects hydrogen peroxide (H_2_O_2_). Strong ROS accumulation was detected in rice cells surrounding 70% of infection sites of Δ*ebg1* as opposed to about 15% of P131 and Δ*ebg1/EBG1* (Fig. 1j, k). Similar ROS accumulation was observed in the barley cells infected by the three strains (Supplementary Fig. 3d, e), indicating that *EBG1* is required to suppress the host ROS burst during plant infection.

To determine whether ROS accumulation is a key factor in reducing the virulence of Δ*ebg1*, we tested whether invasive growth of the mutant could be remediated by addition of diphenyleneiodonium (DPI), an inhibitor of flavoenzymes including NADPH oxidase, a key enzyme for ROS generation. DPI treatment rescued the multi-branching phenotype of IH at 45% infection sites formed by Δ*ebg1* at 36 hpi on rice leaf sheath cells, which was significantly higher than the control experiment (21.7%) carried out with DMSO, which was used as DPI solvent (Fig. 1l, m). The suppression of ROS generation was therefore able to restore impaired IH growth of Δ*ebg1* mutants, albeit not to the level of P131 (58.3%) or the complemented Δ*ebg1/EBG1* strain (56.7%). Similarly, DPI treatment partially rescued the invasive growth of Δ*ebg1* in barley epidermal cells (Supplementary Fig. 3f, g). Therefore, host ROS accumulation is an important factor but not the only reason for impaired biotrophic growth of Δ*ebg1* mutants.

### *M. oryzae* Ebg1 is a secreted protein

**Fig. 2:**
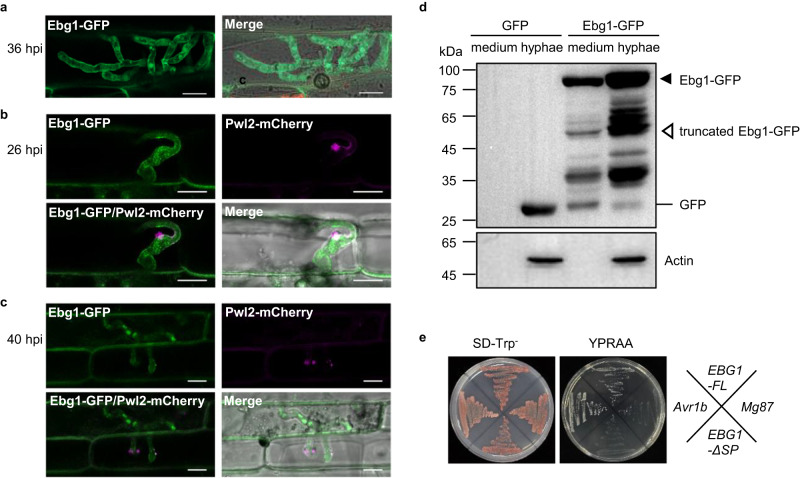
Ebg1 is a secreted protein. **a** Subcellular localization of Ebg1-GFP in the infection hyphae of *M. oryzae*. The Ebg1-GFP fluorescence from the Δ*ebg1*/*EBG1* complemented transformants was distributed mainly around the cell wall and the tip of infection hyphae. Images were captured at 48 h post inoculation (hpi) on barley epidermal leaves. Scale bars = 20 μm. **b**, **c** Laser confocal micrographs of *M. oryzae* strain expressing Ebg1-GFP and Pwl2-mCherry. The Ebg1-GFP/Pwl2-mCherry image shows the overlay of the GFP channel and mCherry channel. A merged image shows the overlay of GFP channel, mCherry channel, and the brightfield channel. Images were captured at 26 hpi (**b**) and 40 hpi (**c**) on rice cultivar LTH. Scale bars = 10 μm. **d** The Ebg1 protein was detected both in vegetative hyphae and cultured medium. A Δ*ebg1*/*EBG1* complemented transformant and a P131/*GFP* transformant were cultured in liquid CM, and total proteins extracted from the mycelia and from the culture filtrates were immunoblotted with an anti-GFP antibody. Intact and truncated versions of Ebg1-GFP were labeled with a black solid triangle and an open triangle, respectively. Anti-actin antibody were used to confirm the protein samples of vegetative hyphae. **e** Ebg1 has a functional signal peptide directing its secretion. The *EBG1* full-length sequence (*EBG1-FL*) and signal peptide deleted sequence (*EBG1-*ΔSP) were constructed into the pSUC2 vector and transformed into yeast YTK12(SUC2-) for the invertase secretion test. The yeast transformants with *EBG1-FL* grew well on the YPRAA medium containing raffinose and antimycin A, whereas *EBG1-*Δ*SP* failed to grow on the same medium. Yeast strains with *Avr1b* and *Mg87* were used as positive and negative controls, respectively.

To determine the subcellular localization of Ebg1 protein, we generated an *EBG1*-*GFP* construct and expressed it in a Δ*ebg1* mutant. Fifteen transformants were obtained, and all recovered wild-type mycelial growth on CM medium supplemented with CFW or CR (Supplementary Fig. 4a), indicating that Ebg1-GFP is a functional chimeric protein. Using one of these transformants, we then investigated the subcellular distribution of the Ebg1-GFP signal in vegetative hyphae, conidia, appressoria, and infection hyphae. In vegetative hyphal cells, the fluorescent signals were present at the hyphal tip, septa, and around the cell wall whereas signals were also unevenly aggregated in conidia, germinated conidia and appressoria, most likely in the endoplasmic reticulum (Supplementary Fig. 4b). In infection hyphae, Ebg1-GFP signals were mainly distributed around the cell wall (Fig. 2a) and showed a distinct pattern of localization compared to the cytoplasmic effector Pwl2. We generated a strain expressing Ebg1-GFP and Pwl2-mCherry and infected rice seedlings. At 26 hpi, Pwl2-mCherry was observed in the biotrophic interfacial complex (BIC), whereas Ebg1-GFP was present in the cell wall of invasive hyphae. A similar pattern of localization of Ebg1-GFP was observed at 40 hpi, with Pwl2-mCherry localizing at nascent BICs in newly invaded cells (Fig. 2b, c). These results suggested that Ebg1 is likely a secreted cell wall and apoplastic protein. To test this idea, we measured Ebg1 in the filtrate and mycelium of liquid CM cultures using immunoblot analysis. As shown in Fig. 2d, two main Ebg1-GFP bands were detected in culture filtrates as well as in mycelium by an anti-GFP antibody, one of which was the molecular weight as expected for the full-length Ebg1-GFP fused protein, and the other was likely a processed form of the Ebg1-GFP. We also validated the activity of the Ebg1 signal peptide (SP) sequence predicted by SignalP using a yeast secretion assay. We observed that the *EBG1* full-length sequence (*EBG1-FL*) but not the SP-deleted sequence (*EBG1-∆*SP) fused to SUC2 was able to deliver invertase into the growth medium with raffinose, as shown in Fig. 2e. When considered together, these data indicate that Ebg1 is a secreted protein.

### Ebg1 is an exo-β-1,3-glucanase important for the evasion of plant basal immunity

**Fig. 3:**
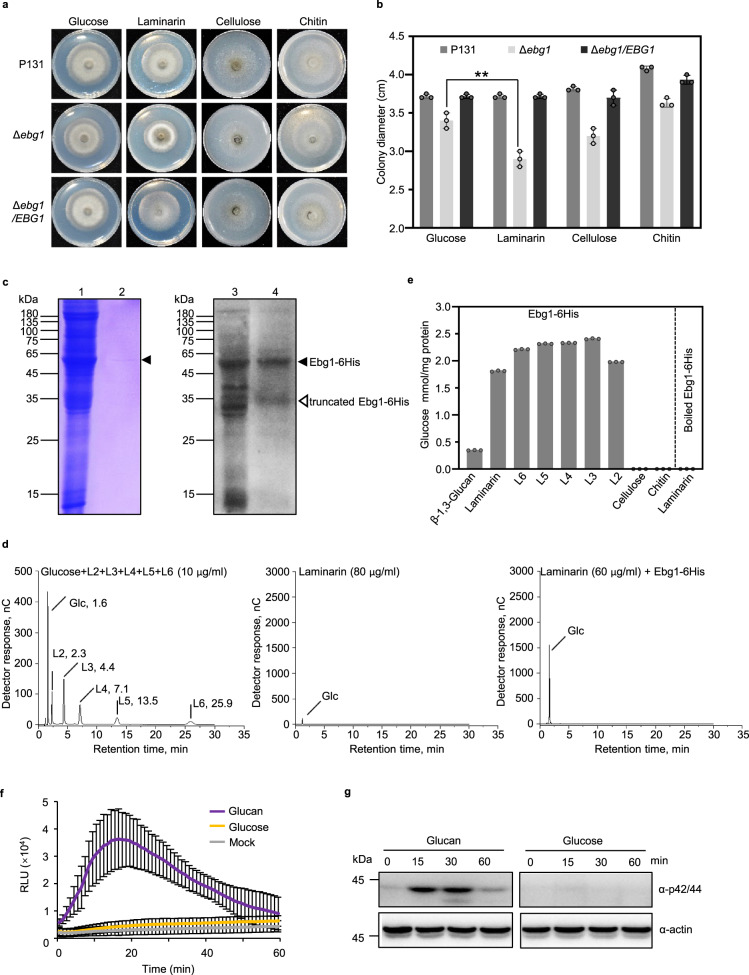
*M. oryzae* Ebg1 is an exo-β-1,3-glucanase. **a**, **b** The growth of the Δ*ebg1* mutant on minimal medium with laminarin is significantly reduced compared to that on minimal medium with other polysaccharides as sole carbon sources. P131, Δ*ebg1* mutant, and Δ*ebg1*/*EBG1* were cultured on minimal medium supplemented with 1% (w/v) glucose, laminarin, cellulose, or chitin at 28 °C for 5 days, photographed (**a**) and measured for colony diameter (**b**). Laminarin, water-soluble β-1,3-linked glucans with occasional β-1,6-linked branches; Cellulose, β-1,4-linked glucans; Chitin, β-1,4-linked N-acetylglucosamine polymers. Error bars denote standard deviations from three biological replicates. **p < 0.01 using Student’s *t* test. Source data with statistic analysis are provided in a Source Data file. **c** Purification of Ebg1-6His protein from the CM culture filtrates of *M. oryzae*. The crude proteins from liquid CM culture filtrates of an *EBG1-6His* strain were purified with ion-exchange chromatography and polyhistidine binding resin before subjected to SDS–PAGE analysis with CBB staining (left panel) and immunoblot analysis using the anti-His antibody (right panel). Lanes 1 and 3: 10 μg of crude proteins; lanes 2 and 4: 0.5 μg and 0.25 μg of purified Ebg1-6His protein. The black solid triangles indicate the correct size for intact Ebg1-6His protein at around 55 kDa, and the open triangle indicates the truncated protein. **d** Purified Ebg1-6His protein hydrolyzed laminarins into glucose. Left panel: laminarioligosaccharides with different lengths (L2–L6) and glucose were loaded on HPLC as standard substances. Middle panel: untreated laminarin were loaded as blank control, and glucose was barely detected. Right panel: laminarin treated with purified Ebg1-6His protein were loaded for HPLC analysis and only glucose was detected from the reaction. Glc, glucose; L2, Laminaribiose; L3, Laminaritriose; L4, Laminaritetraose; L5, Laminaripentaose; L6, Laminarihexaose. **e** Purified Ebg1-6His protein hydrolyzed β-1,3-glucan, laminarin, and laminarioligosaccharides, but neither cellulose nor chitin. The hydrolytic activities of Ebg1-6His on different polysaccharides or oligosaccharides were assayed by detecting the amount of released glucose. Boiled Ebg1-6His protein lost its hydrolytic activity on laminarin. Error bars denote standard deviation. **f** ROS burst was triggered by β-1,3-glucan but not glucose in rice. Leaf disks of 4-week-old rice plants were incubated with 20 μg/ml β-1,3-glucan or glucose, and the luminol-based ROS burst was detected for 60 min constantly. RLU, relative light units. Error bars denote standard deviation, *n* = 8. **g** MAPK activation was triggered in rice leaves by β-1,3-glucan but not glucose. Two-week-old rice seedlings were incubated with 40 μg/ml β-1,3-glucan or glucose for indicated time. Activated MAPKs were detected by immunoblotting with the phospho-p42/44 MAPK antibody. The anti-actin blot was used as a loading control. The experiment was repeated three times with similar results.

Since the three well-known GH17 family proteins, Bgl2p, Scw4p, and Scw10p from *S. cerevisiae* exhibit endo-glucanase activity^32–34^, we wondered whether the *M. oryzae* Ebg1 also functions as a glucanase. We first tested the growth of P131 and the *Δebg1* mutant on minimal medium with different carbohydrates as sole carbon sources (Fig. 3a). Vegetative growth of P131 and Δ*ebg1/EBG1* was comparable on plates supplied with glucose, laminarin, cellulose, and chitin (Fig. 3a, b). However, the colony diameter of the Δ*ebg1* mutant was much smaller on minimal medium supplemented with laminarin, compared with that of Δ*ebg1* on other carbohydrate substrates (Fig. 3a, b). These results suggested that Ebg1 is a putative glucanase that can act on laminarin, a carbohydrate composed of β-1,3-glucan and β-1,6-linked branches.

To biochemically characterize Ebg1, an *EBG1-6His* construct driven by the constitutive RP27 promoter was transformed into the Δ*ebg1* mutant. The expressed Ebg1-6His protein was concentrated and affinity-purified from culture filtrates, and validated by SDS–PAGE with coomassie blue staining and immunoblot analysis (Fig. 3c). The purified Ebg1-6His protein was then incubated with laminarin, and hydrolytic products examined by HPLC. As shown in Fig. 3d, glucose was detected as the sole product. Furthermore, when laminarin oligosaccharides of different lengths, e.g., L2, L3, L4, L5, and L6, were incubated with Ebg1-6His, glucose was also the sole hydrolytic product observed (Supplementary Fig. 5a). Therefore, in follow-up tests, the amount of glucose released into the solution was measured to estimate the hydrolytic activity of Ebg1-6His on various polysaccharides. Ebg1-6His showed strong hydrolysis activity on soluble laminarin and laminarin oligosaccharides, with slightly weaker activity on insoluble β-1,3-glucan, but no activity on cellulose and chitin, both of which are β-1,4-linked polymers (Fig. 3e). In addition, we observed that Ebg1-6His lost its hydrolase activity after boiling (Fig. 3e). The optimal pH and temperature of Ebg1-6His were pH 5.0 and 50 °C, respectively (Supplementary Fig. 5b,c).

It has been reported that β-glucan from pathogenic fungi induces PAMP-triggered immunity^13,14^. Since Ebg1 appears to be an exo-β-1,3-glucanase that can release glucose from β-1,3-glucans, this prompted us to investigate whether Ebg1 might protect *M. oryzae* from β-1,3-glucan-mediated triggering plant immune responses. To test this hypothesis, we measured the ability of β-1,3-glucan to induce plant immunity. We found that β-1,3-glucan, but not glucose, is capable of inducing a ROS burst and MAPK activation in rice leaves, as shown in Fig. 3f, g.

Previous studies showed that GH17 family members have two conserved glutamic acid residues^35^, and Ebg1 has these residues at positions 378 and 476 (Supplementary Fig. 6a). To determine whether the two residues are important for Ebg1 activity, we generated an *EBG1* construct in which the two residues were simultaneously replaced by glutamine, named *EBG1*^*E378Q/E476Q*^, and transformed this into the Δ*ebg1* mutant (Supplementary Fig. 6a). Fifteen transformants were obtained, and all of them, including Δ*ebg1/EBG1*^*E378Q/E476Q*^-1 and -6, were similar to Δ*ebg1* in phenotype, exhibiting defects in the utilization of polysaccharides with β-1,3 glycosidic linkage (Supplementary Fig. 6b, c), sensitivity to CFW and CR (Supplementary Fig. 6d, e), and a significant reduction in virulence (Supplementary Fig. 6f, g). We then tested the exo-β-1,3-glucanase activity of purified Ebg1^E378Q/E476Q^-6His protein and found that the mutant protein largely lost enzymatic activity (Supplementary Fig. 6h, i). Taken together, these results provide evidence that *EBG1*^*E378Q/E476Q*^ is unable to rescue the defects of Δ*ebg1* mutants, indicating that Glu-378 and Glu-476 are required for Ebg1 to act as a functional exo-β-1,3-glucanase, which is important for its role in virulence of *M. oryzae*. Additionally, exogenous application of purified protein Ebg1-6His could rescue the infection defect of Δ*ebg1* strain (Supplementary Fig. 7a–d) and suppress the host ROS accumulation triggered by Δ*ebg1* infection (Supplementary Fig. 7e–h), further confirming that Ebg1 plays an important role in suppressing plant basal immunity.

### Ebg1 can act as a PAMP, independent of its enzymatic activity

**Fig. 4:**
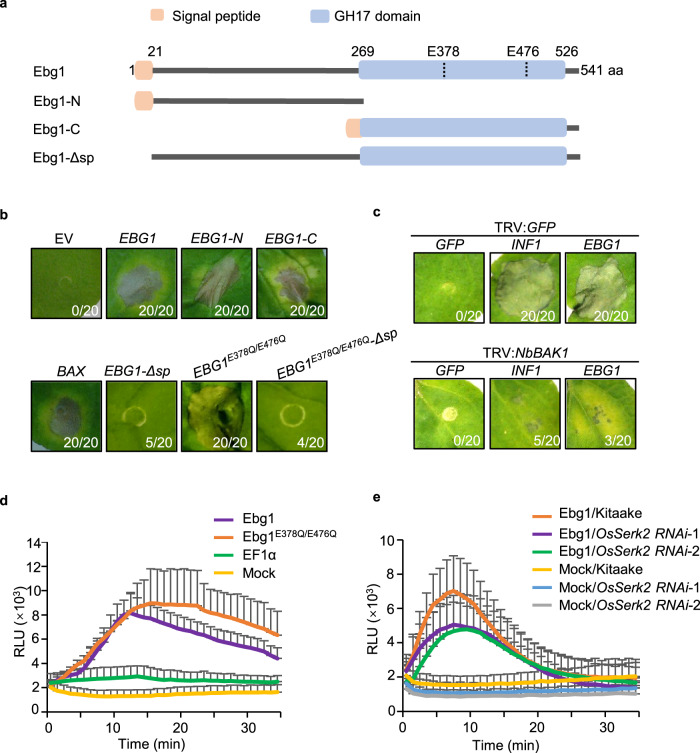
Ebg1 elicits tobacco cell death and activates rice immune responses. **a** Schematic diagram of *M. oryzae* Ebg1 protein. The signal peptide is indicated in orange, and the GH17 hydrolase domain is indicated in blue. Ebg1, Full-length protein; Ebg1-N, the N-terminal half of Ebg1 (1-267aa); Ebg1-C, the signal peptide linked with the C-terminal half of Ebg1 (1-21aa fused to 268-541aa). Ebg1-Δsp, Ebg1 protein without the signal peptide. **b** Ebg1 induced cell death in *N. benthamiana*. Tobacco leaves infiltrated with *Agrobacterium* strains carrying *BAX*, *EBG1*, *EBG1-N*, *EBG1-C*, *EBG1-Δ*sp, *EBG1*^*E378Q/E476Q*^, and *EBG1*^*E378Q/E476Q*^*-Δsp* were photographed at 5-day post infiltration (dpi) and stained with trypan blue. The numbers indicate leaves with cell death versus total treated leaves. EV, empty vector was used as a negative control, and BAX was used as a positive control. **c** Cell death triggered by Ebg1 in *N. benthamiana* requires *NbBAK1*. Tobacco plants were subjected to VIGS by inoculation with TRV:*GFP* or TRV:*NbBAK1*. Three weeks after VIGS treatment, *GFP*, *INF1*, and *EBG1* were transiently expressed in the gene-silenced leaves and then leaves were photographed at 7 dpi. The numbers indicate leaves with cell death versus total treated leaves. **d** Purified Ebg1 protein induced ROS spiking in rice cultivar ZH11. Leaf disks of 4-week-old rice plants were incubated with 80 μg/ml Ebg1-6His or Ebg1^E378Q/E476Q^-6His, and the luminol-based ROS burst was detected for 35 min constantly. Ef1a-6His protein and mock treatment were used as negative controls. RLU, relative light units. Error bars denote standard deviation, *n* = 8. **e** Purified Ebg1 protein induced ROS generation in rice cultivar Kitaake and reduced ROS level in two *OsSerk2* RNAi lines. Leaf disks of 4-week-old rice plants were incubated with 80 μg/ml Ebg1-6His, and the luminol-based ROS burst was detected for 35 min constantly. Mock treatment was used as the negative control. RLU, relative light units. Error bars denote standard deviation, *n* = 6.

Many GH family proteins are known to act as PAMPs, including the GH12 family protein PsXEG1 from *P. sojae*^36^ and the GH18 family protein MoChia1 from *M. oryzae*^26^. Therefore, we reasoned that Ebg1 might also be recognized as a PAMP. Ebg1 contains an N-terminal signal peptide (SP, 1-21aa) and a GH17 domain at the C-terminal region (269-526aa) (Fig. 4a). When full-length *EBG1* was transiently expressed through Agroinfiltration in *N. benthamiana*, it caused conspicuous cell death (Fig. 4b). Since Ebg1 is expressed in both full-length and truncated forms (Figs. 2d and 3c), we tested whether the individual N- and C-terminal truncated Ebg1 proteins can induce cell death. When transiently expressing each truncated protein carrying the signal peptide with either the N-terminus (Ebg1-N, 1-267aa) or C-terminal part of Ebg1 (Ebg1-C, signal peptide 1-21aa fused in-frame to 268-541aa), both the N- and C-terminal truncated proteins induced cell death responses in tobacco leaves (Fig. 4a, b). In addition, the Ebg1^E378Q/E476Q^ mutant also induced cell death responses in tobacco leaves. However, Ebg1 or Ebg1^E378Q/E476Q^ protein without the signal peptide (*EBG1-Δsp* and *EBG1*^*E378Q/E476Q*^*-Δ*sp) failed to trigger a response (Fig. 4a, b). Immunoblotting confirmed that full-length Ebg1, and each mutant or truncated protein were all expressed as expected (Supplementary Fig. 8a). These results provide evidence that Ebg1 can trigger cell death in tobacco leaves independent of its enzymatic activity.

To investigate the basis of cell death observed in the *N. benthamiana* assays, we decided to test the role of BAK1, a key protein kinase in multiple PAMP pathways, which directly interacts with different PAMP receptors^37^. To test the potential role of BAK1 in cell death induced by Ebg1, we silenced the *BAK1* gene of *N. benthamiana* using a tobacco rattle virus (TRV)-based virus-induced gene silencing (VIGS) system before expressing *EBG1* or *INF1* by agroinfiltration^38^. INF1 failed to trigger cell death in *BAK1*-silenced plants (Fig. 4c), consistent with a previous report that BAK1 is required for INF1-induced cell death^37^. Interestingly, Ebg1 was also unable to induce a cell death response in *BAK1*-silenced plants (Fig. 4c). For these experiments, we confirmed that both INF1 and Ebg1 were expressed at the expected size in plants transformed with TRV:*NbBAK1* or TRV:*GFP* by immunoblots (Supplementary Fig. 8b) and that *BAK1* expression was significantly reduced in the TRV:*NbBAK1*-transformed plants compared to the TRV:*GFP*-transformed plants, as revealed by RT-qPCR analysis (Supplementary Fig. 8c). We conclude that *BAK1* is required for Ebg1-triggered cell death in *N. benthamiana*.

Using Ebg1-6His protein purified as described above, we further tested whether Ebg1 can induce rice PAMP responses, such as ROS generation and MAPK activation^39^. When rice leaf disks were supplemented with purified Ebg1-6His and Ebg1^E378Q/E476Q^-6His, ROS generation and MAPK activation were clearly observed (Fig. 4d and Supplementary Fig. 8d), confirming that the elicitor activity of Ebg1 in rice plants is independent of its enzymatic activity.

The rice genome contains two *BAK1* homologs *OsSerk1* and *OsSerk2*, which are key regulators in rice development and immunity^40–42^. We also observed that the Ebg1-6His-induced ROS burst was impaired in RNAi rice lines of *OsSerk2* as compared to the wild type cultivar (Fig. 4e), suggesting that *OsSerk2* contributes to the Ebg1-induced rice immunity responses.

### Ebg1 interacts with translation elongation factor 1 alpha protein to evade plant immunity

**Fig. 5:**
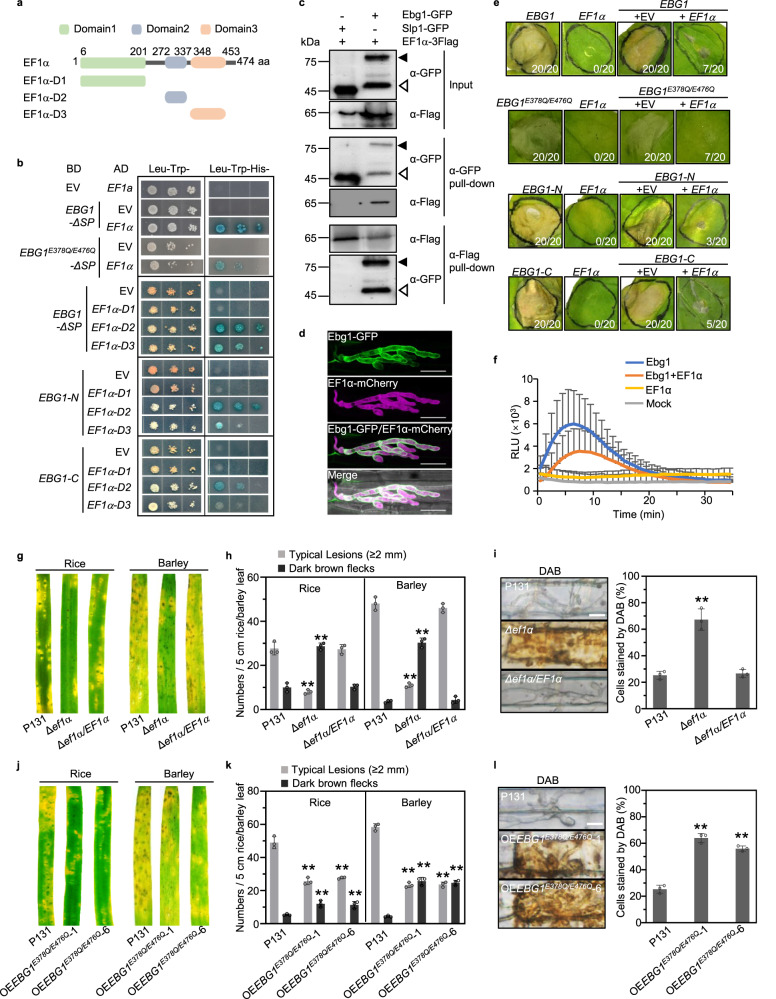
*M. oryzae* EF1α interacts with Ebg1 and prevent plant immune responses triggered by Ebg1. **a** Schematic diagram of *M. oryzae* EF1α protein. Domain 1 with putative GTP binding activity, Domain 2 and Domain 3 with beta-barrel structure, are indicated in green, gray, and orange boxes, respectively. EF1α-D1, EF1α-D2, EF1α-D3 are truncations of EF1α protein with indicated domains. **b** The yeast two-hybrid assays showing that EF1α-D2 and EF1α-D3, but not EF1α-D1 interact with Ebg1, Ebg1^E378Q/E476Q^, Ebg1-N, and Ebg1-C. **c** Co-immunoprecipitation assays showing that EF1α interacts with Ebg1 in the extracellular matrix of *M. oryzae*. The *EBG1-GFP* and *EF1α-3Flag* vectors were co-transformed into *M. oryzae* P131 for Co-immunoprecipitation assay. Total proteins from liquid CM culture filtrates of the transformant were subjected to GFP or Flag pull-down analysis. Total proteins or Co-IP elutions were detected by anti-GFP antibody or anti-Flag antibody. The P131 strain expressing *SLP1-GFP* and *EF1α-3Flag* was used as a negative control. The solid and unfilled triangles point to the signals for intact Ebg1-GFP protein and the truncated Ebg1-GFP protein, respectively. **d** Co-localization of Ebg1-GFP and EF1a-mCherry in the apoplast of invasive hyphae of *M. oryzae*. An *M. oryzae* strain expressing Ebg1-GFP and EF1a-mCherry was used to inoculate rice sheath. The Ebg1-GFP/EF1a-mCherry image shows the overlay of GFP channel and mCherry channel. The merged image shows the overlay of GFP channel, mCherry channel, and the brightfield channel. Images were captured at 32 hpi on rice cultivar LTH. Scale bars = 20 μm. **e** *EF1α* inhibited Ebg1-triggered cell death in *N. benthamiana*. Cell death was observed in tobacco leaves infiltrated with *Agrobacterium* strains individually carrying *EBG1*, *EBG1*^*E378Q/E476Q*^, *EBG1-N*, or *EBG1-C*, but not in the leaves co-expressing *EF1α* with *EBG*1, *EBG1*^*E378Q/E476Q*^, *EBG1-N*, or *EBG1-C*. Pictures are representative tobacco leaves photographed at 7-day after agroinfiltration, and numbers indicate leaves with cell death versus total treated leaves. EV, empty vector. **f** Purified Ef1α protein partially blocks the Ebg1-induced ROS spiking in rice cultivar ZH11. Leaf disks of 4-week-old rice plants were incubated with 80 μg/ml Ebg1-6His with or without Ef1a-6His protein, and the luminol-based ROS burst was detected for 35 min constantly. Ef1a-6His protein alone and mock treatment were used as negative controls. RLU, relative light units. Error bars denote standard deviation, *n* = 8. **g** Δ*ef1α* mutants are significantly reduced in virulence on rice and barley. Rice and barley leaves were spray-inoculated with the conidial suspensions (3 × 10^4^ spores/ml) of P131, Δ*ef1α* mutant, and complemented transformant Δ*ef1α*/*EF1α*, and photographed at 5 dpi. **h** Higher ratios of dark brown spots were formed on rice and barley leaves by Δ*ef1α* mutant than by P131. Numbers of typical lesions and dark brown spots on rice and barley leaves were counted per leaf. **i** Δ*ef1α* mutants fail to prevent ROS production. Rice sheath cells drop-inoculated with conidia suspensions (1 × 10^5^ spores/ml) of P131, Δ*ef1α*, and Δ*ef1α*/*EF1α* strains were stained with DAB at 30 hpi, and the percentage of infection sites with DAB-stained cells was calculated. Scale bars = 20 μm. **j** Overexpression of *EBG1*^*E378Q/E476Q*^ in P131 driven by the *EF1α* promoter (*OEEBG1*^*E378Q E476Q*^) resulted in reduced virulence on rice and barley leaves. Rice and barely leaves were spray-inoculated with conidial suspensions (5 × 10^4^ spores/ml) of P131 and *OEEBG1*^*E378Q/E476Q*^ transformants. The inoculated leaves were photographed at 5 dpi. **k** Quantitative analysis of lesion formation on rice and barley leaves by P131 and OE*EBG1*^*E378Q/E476Q*^ transformants. Numbers of typical disease lesions and dark brown spots on rice and barley leaves were counted per leaf. **l** OE*EBG1*^*E378Q/E476Q*^ transformants induced ROS production. Rice sheath cells drop-inoculated with conidial suspensions (1 × 10^5^ spores/ml) of P131 or OE*EBG1*^*E378Q/E476Q*^ transformants were stained with DAB at 30 hpi and the percentages of the DAB stained infection sites were calculated. Scale bars = 20 μm. For all the above statistics, error bars denote standard deviations from three biological replicates. ** and * indicate *p* < 0.01 and *p* < 0.05 significant differences compared with corresponding WT controls. One-way ANOVA with post-hoc Turkey tests were used in (**h**), (**i**), (**k**) and (**l**). Source data with statistic analysis are provided in a Source data file.

To investigate how *M. oryzae* evades Ebg1-triggered host innate immunity during infection, we searched for proteins that interact with Ebg1 by yeast two-hybrid analysis (Y2H). Using signal peptide-deleted coding sequence of *EBG1* (*EBG1-∆*SP) as the bait, we obtained nine putative Ebg1-interacting proteins from a cDNA library constructed with *M. oryzae*-infected rice leaves (Supplementary Table 1). Interestingly, among the Ebg1-interacting proteins was an elongation factor 1 alpha (EF1α) encoded by *MGG_03641*, which contains three EF-Tu domains: domain 1 for GTP binding, domains 2 and 3 with beta-barrel structures (Fig. 5a). Ebg1 was confirmed to interact with EF1α but not with the other two closely related EF-Tu domain-containing proteins, MGG_08162 and MGG_02504, by the Y2H assay (Supplementary Figs. 9, 10a, b). In addition, EF1α interacts with Ebg1 and the loss-of-enzyme activity mutant Ebg1^E378Q/E476Q^ via its domain 2 and domain 3 in a Y2H assay (Fig. 5b).

A co-immunoprecipitation (Co-IP) assay showed that Ebg1 and EF1α could be immunoprecipitated by each other from filtrates of liquid CM cultures of a transgenic *M. oryzae* expressing *EBG1-GFP* and *EF1α-3Flag* (Fig. 5c). We also collected apoplastic fluid of blast fungus-infected barley leaves and detected both Ebg1-GFP and EF1α-3Flag in the fluid (Supplementary Fig. 10c), providing evidence that EF1α-3Flag protein can be secreted into the apoplast during *M. oryzae* infection. Further mass spectrometry analyses identified multiple peptides of Ebg1 and Ef1α in the filtrates of P131 liquid medium culture (Supplementary Data 1) and two peptides of Ef1α in the apoplastic fluid of barley leaves infected with P131 (Supplementary Data 2). Further, we generated transgenic *M. oryzae* co-expressing EBG1-GFP and EF1α-mCherry to check whether the two proteins are co-localized during infection. After inoculation of rice leaves with this transgenic *M. oryzae* strain, we observed that the fluorescence signals of Ebg1-GFP and EF1α-mCherry were partially co-localized in the apoplastic space of invasive hyphae (Fig. 5d). These lines of evidence indicate that Ebg1 interacts and co-localized with EF1α in the apoplast during invasive growth of infection hyphae.

To understand the biological relevance of the interaction between Ebg1 and EF1α, we transiently co-expressed *EF1α* and *EBG1* or *EF1a* and *EBG1*^*E378Q/E476Q*^ in *N. benthamiana*. Surprisingly, cell death was not observed in tobacco leaves in these co-expression assays (Fig. 5e), indicating that the presence of EF1α is sufficient to suppress the elicitor activity of Ebg1 and Ebg1^E378Q/E476Q^. Immunoblotting confirmed that full-length Ebg1, and Ebg1^E378Q/E476Q^ were expressed as expected (Supplementary Fig. 10d). Using a bimolecular fluorescence complementation (BiFc) assay, we also observed that Ebg1-nYFP interacted with cYFP-EF1α in the apoplast of tobacco leaf cells when they were transiently co-expressed (Supplementary Fig. 10e–g). Furthermore, we verified that both the N and the C-terminal regions of Ebg1 could interact with the domain 2 and domain 3 of the EF1α protein in a Y2H assay and that EF1α also inhibited the elicitor activity of the Ebg1-N and Ebg1-C truncated proteins (Fig. 5b, e, Supplementary Fig. 10d).

To further investigate the effect of the presence of EF1α, we tested whether PAMP responses can be induced by purified Ebg1-6His protein in tobacco leaves that transiently expressed EF1α. The Ebg1-6His-induced MAPK activation was not detected in tobacco leaves pre-expressing EF1α (Supplementary Fig. 11a). These data provide evidence that *M. oryzae* EF1α has the capacity to prevent Ebg1 from triggering plant immunity in tobacco leaves.

In order to test whether Ef1α has an effect on Ebg1-induced rice immune responses, we purified Ef1α-GFP-6His protein from yeast strains (Supplementary Fig. 11b) and tested its effects using purified Ebg1 proteins. Using anti-GFP beads, the Ef1α-GFP-6His protein could pull down Ebg1-GST protein in vitro (Supplementary Fig. 11c). The purified Ef1α-GFP-6His protein did not inhibit the hydrolysis activity of Ebg1-6His on laminarin (Supplementary Fig. 11d), but significantly suppressed Ebg1-induced ROS generation on rice leaves (Fig. 5f). These data suggest that purified EF1 α protein prevents Ebg1 from triggering plant immunity in rice leaves.

### *M. oryzae EF1*α is important for virulence

The putative role of EF1α in suppressing the PAMP activity of Ebg1 suggested that *EF1α* may be important for virulence of *M. oryzae*. To test this idea, we generated Δ*ef1α* mutants in the *M. oryzae* P131 background (Supplementary Fig. 12a,b). The Δ*ef1α* mutants grew normally on OTA plates (Supplementary Fig. 12c, d), suggesting that *EF1α* is dispensable for normal mycelial growth and development of *M. oryzae*. However, the Δ*ef1α* mutants were notably reduced in their ability to cause blast disease in both rice and barley seedlings (Fig. 5g, h). Furthermore, the Δ*ef1α* mutants also induced a ROS burst in rice leaf sheath cells (Fig. 5i) and barley epidermal cells (Supplementary Fig. 12e). EF1α is therefore important for virulence of *M. oryzae*.

We reasoned that if Ebg1 interacts with EF1α to evade recognition as a PAMP, then this might require an excess of EF1α over Ebg1. To test this idea, we therefore generated an overexpression construct OE*EBG1*^*E378Q/E476Q*^ using the promoter of *EF1α* and transformed this into the wild-type *M. oryzae* strain P131. The resulting transformants showed consistently reduced virulence on rice and barley plants (Fig. 5j, k) and induced a ROS burst in cells surrounding infection sites (Fig. 5l and Supplementary Fig. 12f). Together, these data further suggest that Ebg1 is a PAMP that does not require its enzymatic activity to induce plant immunity and that *M. oryzae* normally suppresses its PAMP activity in a manner that requires EF1α.

In addition, a pBLAST search suggested that orthologues of both Ebg1 and EF1α are widely distributed among fungi (Supplementary Fig. 1 and Supplementary Fig. 9). However, Ebg1 orthologues from different fungi are diversified in sequence while EF1α orthologues are highly conserved (Supplementary Figs. 1,  9, 13a,b). Structural prediction indicates that Ebg1 from *M. oryzae* is highly similar to Ebg1 from *Fusarium graminearum* (Supplementary Fig. 13c), suggesting that Ebg1 orthologues in different fungi may play similar roles. To test this idea, we performed Y2H assays, which showed that the Ebg1 and EF1α orthologues from *F. graminearum* could interact with each other, and that *M. oryzae* Ebg1 could also interact with *F. graminearum* EF1α, and vice versa (Supplementary Fig. 13d). Furthermore, Ebg1 and EF1α orthologues from *F. graminearum* could largelly rescue the mutant phenotypes of Δ*ebg1* and Δ*ef1α* mutants of *M. oryzae*, respectively (Supplementary Fig. 13e, f). Together, these data suggest that the interaction between Ebg1 and EF1α in plant pathogenic fungi may be a conserved mechanism whereby fungal pathogens evade plant immunity and safeguard cell wall remodeling during infection.

## Discussion

**Fig. 6:**
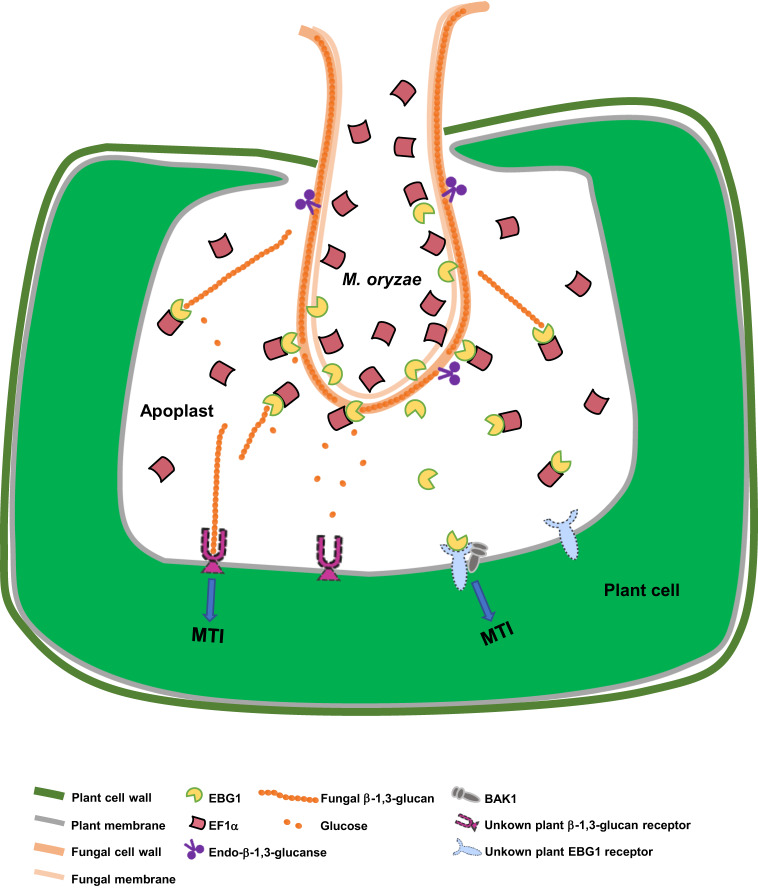
A working model for the potential mechanism by which Ebg1 and EF1α act to evade host plant immunity during invasive hyphal growth by *M. oryzae*. Fungal β-1,3-glucan fragments released by endo-β-1,3-glucanases at the fungal-plant interface can be perceived by a specific but unknown plant β-1,3-glucan receptor, and lead to PAMP-triggered plant immunity (PTI). *M. oryzae* expresses and secretes Ebg1 as an exo-β-1,3-glucanase hydrolyzing β-1,3-glucans into glucose to prevent the induction of PTI. Meanwhile, Ebg1 itself is a PAMP recognized by an unknown plant Ebg1 receptor to activate PTI. *M. oryzae* expresses an excess of EF1α protein, which is recruited by Ebg1, to evade the recognition of Ebg1, consequently preventing PTI.

This study reports evidence that *M. oryzae* cell wall remodeling of β-1,3-glucan during plant infection involves at least two rounds of PTI and counter-PTI between the pathogen and host plant (Fig. 6). In the first round, plant cells recognize fungal β-1,3-glucans that are likely released by endo-β-1,3-glucanases during cell wall remodeling for invasive hyphal growth, consequently, initiating the first PTI to limit fungal colonization^14,43^ (Fig. 3f, g). To evade β-1,3-glucan-triggered plant immunity, *M. oryzae* secretes Ebg1, an exo-β-1,3-glucanase, to hydrolyze fungal-released β-1,3-glucans into glucose (Fig. 3d, e, Supplementary Fig. 5). As a response, the plant host perceives Ebg1 as a PAMP with an unknown receptor to activate the second PTI (Fig. 4b–e). *M. oryzae* then appears to utilize an EF1α protein that interacts and co-localizes with Ebg1 to evade Ebg1-triggered PTI (Fig. 5b–f). By evolving these two forms of counter-PTI mechanisms, *M. oryzae* is capable of protecting its cell wall from being recognized during infection without being detected by immune receptor(s) of host plants, thereby ensuring efficient colonization of plant tissues. Orthologues of both Ebg1 and EF1α are widely distributed among fungi (Supplementary Figs. 1, 9). Our study further showed that Ebg1 and EF1α orthologues from *F. graminearum* could also interact with each other and could rescue the mutant phenotypes of Δ*ebg1* and Δ*ef1α* mutants in *M. oryzae*, respectively (Supplementary Fig. 13). Therefore, the interaction between Ebg1 and EF1α may be a conserved mechanism of fungal pathogens to evade β-1,3-glucan-triggered host immunity during infection.

The *M. oryzae* Ebg1 identified in this study is an exo-β-1,3-glucanase of the GH17 family in fungi. Biochemically, Ebg1 is active on linear β-1,3-glucans and laminarin and produces only glucose as the end product (Fig. 3d, e). Biologically, Ebg1 is important for *M. oryzae* to maintain cell wall integrity and cause blast disease (Fig. 1a–e). Furthermore, Ebg1 triggers plant immune responses in a manner that is independent of its hydrolase activity (Fig. 4b, d). These characteristics are distinct from that of CfGH17-1 in *Cladosporium fulvum*^44^, which is the sole β-1,3-glucanase of GH17 family characterized so far in filamentous fungal pathogens. CfGH17-1 is likely an endo-β-1,3-glucanase of the GH17 family that induces plant cell death depending on its enzymatic activity; however, it is not required for virulence by *C. fulvum*^44^. Ebg1 is also distinct from previously identified Scw4p, Scw10p, Scw11p and Bgl2p of *S. cerevisiae*^32–34^. Although these yeast proteins of the GH17 family function in cell wall remodeling, they are all endo-β-1,3-glucanases. Furthermore, Ebg1 also showed no similarity in amino acid sequence to previously reported exo-β-1,3-glucanases of the GH5 family, such as Exg1 in *S. cerevisiae*^45–47^ and *Candida albicans*^48^, GLU1 in *Pyrenophora tritici-repentis*^49^, and Exo1 in *Pythium insidiosum*^50^, which are all important in cell wall remodeling. Interestingly, Exo1 is characterized as an intracellular immunoreactive protein triggering human cell antibody responses^51^, while *M. oryzae* Ebg1 is perceived by plant cells in the apoplast, suggesting that mammalian and plant host cells may have independently evolved mechanisms to recognize exo-β-1, 3-glucanase as a PAMP.

Several previous reports have shown that GH proteins from filamentous plant pathogens can induce host immune responses without requiring enzymatic activity, including the GH11 member EIX from *T. viride*^52^, the GH12 member PsXEG1 from *Phytophthora sojae*^36^ and the GH18 member MoChia1 from *M. oryzae*^26^. However, it remains largely unknown how fungal pathogens subvert perception of GH proteins by host plants. This study shows that Ebg1 can trigger plant immune responses, independent of its hydrolase activity (Fig. 4b, d), and that EF1α can interact with both the N and C termini of Ebg1 and that this interaction prevents the triggering of cell death in transient co-expression assays in tobacco (Fig. 5b–e). The interaction between Ebg1 and EF1α may therefore be the means by which the PAMP activity of Ebg1 is prevented during infection. Interestingly, EF1α has overall similarity to EF-Tu from *Escherichia coli*^53,54^ and EF-Tu-a from *Acidovorax avenae*^55^ (Supplementary Fig. 9), which are well-known bacterial PAMPs. *E. coli* EF-Tu contains the elf18 peptide with an acetyl-xKxKFxR motif crucial for inducing defense response in *Brassicaceae* species^53^, while the EFa50 from the Lys176 to Gly225 region of *A. avenae* EF-Tu-a could mount defense responses in rice^55^. However, EF1α lacks these motifs (Supplementary Fig. 14), consistent with its inability to induce cell death in tobacco plants (Fig. 5e). The presence of EF1α appears to be necessary for *M. oryzae* to evade Ebg1-induced plant immune responses, but it remains unclear how these two proteins interact within the apoplast to evade Ebg1-triggered PTI. Further studies will therefore be required to identify a plant pattern recognition receptor that recognizes Ebg1 and to determine how this perception is prevented, or inhibited by the presence of EF1α.

## Methods

### Strains and culture conditions

*M. oryzae* P131^31,56^ was used as a wild-type strain to generate all mutants in this study (Supplementary Table 2). All *M. oryzae* wild-type strain and mutants were propagated on oatmeal agar medium (OTA) plates. Cell wall integrity was tested on complete medium (CM)^57^ with 200 μg/ml CR and 100 μg/ml CFW, whereas the sugar utilization was examined on a minimal medium with 1% (w/v) glucose, laminarin, cellulose or chitin. Conidia were prepared as previously reported^56^. For *M. oryzae* inoculation, rice (*Oryza sativa* cv LTH or NBT) plants were grown at 28 °C for 2–4 weeks, barley (*Hordeum vulgare* cv E9) plants were grown at 28 °C for 5–7 days. Tobacco (*Nicotiana benthamiana*) plants were grown at 25 °C for 4 weeks before agroinfiltration or PAMP treatment.

### Gene knockout and complementation

*EBG1* and *EF1α* knockout mutants were generated as reported^56^ with the primers listed in Supplementary Table 3. Protoplasts were isolated and transformed by the PEG/CaCl_2_ method^58^. The mutants were first screened by PCR to detect flanking regions of hygromycin marker and then confirmed by Southern blot analysis. Probes used for Southern blot were labeled with the DIG High Prime DNA Labeling and Detection Starter Kit II (Roche). For complementation, the full-length *EBG1* gene or *EF1α* containing a 1.5 kb native promoter fragment were amplified and cloned into pGTN^59^, and the resulting constructs were transformed into protoplasts of corresponding mutants. At least 15 neomycin-resistant complemented transformants for Δ*ebg1/EBG1* or Δ*ef1α/EF1α* were picked up for functional tests, and the data from one complemented strain each were presented. The *EBG1*^*E378Q/E476Q*^ mutation was generated using PCR-based mutagenesis with the Q5 Site-Directed Mutagenesis Kit (NEB), and *EF1a* promoter was used as the overexpression promoter to drive *EBG*1^*E378Q/E476Q*^ gene expression for functional tests.

### Virulence test and infection process observation

To measure the virulence of *M. oryzae* strains, conidial suspensions at a concentration of 5 × 10^4^ conidia/ml in 0.025% Tween 20 were sprayed on rice or barley leaves. The inoculated leaves were incubated in a moist, dark chamber at 28 °C for 30 h and then under regular illumination^56^. Disease lesions were scored and photographed at 5 day post-inoculation (dpi). To investigate the infection process of *M. oryzae* strains, 3-week-old rice sheaths were injected with conidial suspensions of 1 × 10^5^ conidia/ml in 0.025% Tween 20 and incubated in a moist, dark chamber at 28 °C. Microscopy observations were performed at 18, 24, 30, 36 h post-inoculation (hpi) under a Nikon 90i microscope.

### DAB staining and exogenous DPI and Ebg1 treatments

Host-derived ROS was detected by staining rice sheaths with 3,3’-diaminobenzidine (DAB, Sigma-Aldrich). Rice sheaths or barley leaves inoculated with *M. oryzae* at 30 hpi, were immersed in 1 mg/ml DAB solution (pH 3.8) for 8 h and then de-stained overnight with the clearing solution (ethanol: acetic acid = 94: 4, v/v) before microscopic observation^60^. For assessing invasive growth of *M. oryzae* and ROS accumulation in rice sheath or barley leaves by the treatment of 0.5 μM diphenyleneiodonium (DPI)^59^ or Ebg1-His, drop inoculation of *M. oryzae* conidial suspension was used, and DPI and Ebg1-His were added on the drop inoculation site at 12 hpi and in the conidial suspension, respectively.

### Subcellular localization of Ebg1 and co-localization of Ebg1 with EF1α

A complemented strain Δ*ebg1*/*EBG1* expressing Ebg1-GFP was used to determine the subcellular localization of Ebg1, and a strain expressing Ebg1-GFP and Pwl2-mCherry was used to observe the localization of Ebg1 and Pwl2. A Nikon A1 laser scanning confocal microscope was used to observe the fluorescence distribution in vegetative hyphae cultured in liquid CM, conidia (0 h), conidial germ tubes (2 h), appressoria (24 h) on hydrophobic slides, and invasive hyphae of infected rice sheath cells at 20 and 40 hpi.

To construct plasmid EF1α-mCherry with its native promoter for observing its co-localization with Ebg1, the EF1a coding sequence with native promoter was amplified using primers 3641-Pro-F and 3641-mCh-R and *M. oryzae* genomic DNA as template, and mCherry was amplified with primers mCh-Hind-F and mCh-Kpn-R. The resulting fragments were inserted into the SacII and KpnI digested site in pCB1532 by In-Fusion cloning. The plasmid EF1α-mCherry was then transformed into an *M. oryzae* strain expressing Ebg1-GFP. Positive transformants were selected by sulfonylurea resistance. Laser confocal microscopy was carried out to check the fluorescence of the transformants using a Leica SP8 laser confocal microscope.

### Yeast invertase secretion assay

The yeast signal sequence trap vector pSUC2T7M13ORI (pSUC2)^61^, which carries a truncated invertase gene lacking signal peptide (SP), was used in this assay. The full-length *EBG1* sequence (*EBG1-FL*) and SP-deleted coding sequences (*EBG1-∆*SP) were independently cloned into pSUC2 (Supplementary Table 3). The pSUC2-derived plasmids (0.5 µg) were transformed into the invertase-deficient yeast strain YTK12 (SUC2-) using the PEG/LiAc transformation method. Transformants were selected on yeast minimal tryptophan dropout medium (CMD-W medium, 0.67% yeast N base without amino acids, 0.075% tryptophan dropout supplement, 2% sucrose, 0.1% glucose, and 2% agar). Yeast colonies were then plated onto YPRAA plates (1% yeast extract, 2% peptone, 2% raffinose, and antimycin A at 2 µg/l) to detect the invertase secretion, Avr1b and Mg87 were used as the positive and negative controls, respectively.

### Purification of Ebg1 or Ebg1^E378Q/E476Q^ protein from *M. oryzae* liquid medium

For protein purification, the coding sequence for six contiguous histidine residues was fused in-frame after the C-termial sequence of *EBG1* or *EBG1*^*E378Q/E476Q*^ gene (Supplementary Table 3). The PCR product was ligated into the pRTN expression vector, and the resulting construct pRTN-*EBG1*-*6His* transformed into a Δ*ebg1* mutant. A neomycin-resistant complemented transformant was used for protein expression and purification, which was cultured in liquid culture medium with 0.6% yeast extract, 0.3% casein enzymatic hydrolysate, 0.3% casein acids hydrolysate, and 1% sucrose at 28 °C, 160 rpm for 36 h. The culture was first filtrated with cheesecloth, and the filtrates concentrated by ultrafiltration (Vivaflow 50; Sartorius Stedim Biotech, Aubagne, France and Amicon Ultra-15; Millipore, Billerica, USA. 30 kDa) and equilibrated with 20 mM PBS buffer (pH 8.0) containing 150 mM NaCl^62,63^. Then, crude proteins were further purified with ion-exchange chromatography (IEC) and polyhistidine binding resin (TALON metal affinity resin; Clontech, CA, USA) with the PBS buffer. The resin was sequentially washed with 80 mM imidazole solution buffer containing 20 mM PBS (pH 8.0) and 150 mM NaCl. Finally, bound Ebg1-6His protein was eluted with a 300 mM imidazole solution buffer containing 20 mM PBS (pH 8.0) and 150 mM NaCl. The eluate was concentrated and equilibrated with 20 mM PBS buffer (pH 8.0) containing 150 mM NaCl by ultrafiltration (Amicon Ultra-4; Millipore, Billerica, USA. 30 kDa). The crude and purified Ebg1-6His proteins were subjected to SDS–PAGE followed by CBB staining and immunoblot analysis using an anti-His antibody^63^. Protein concentration was determined using a NanoDrop (NanoDrop One) or using a Bradford protein assay kit (Thermo-Fisher Scientific) with BSA (Sigma-Aldrich) as a standard.

### High-performance liquid chromatography (HPLC) analysis

A Dionex ICS3000 system equipped with a pump, and pulsed amperometric detector, an automated sampler with a 25 μl injection loop, and a chromeleon chromatography management system (Dionex, Sunnyvale, CA) was used for sugar identification and quantification. The analytical CarboPac PA10 pellicular anion-exchange resin column (250 × 4 mm) preceded by a CarboPac PA10 guard column (50 × 4 mm) was used for sugar separation. The glucose was eluted with 25 mM NaOH at a flow rate of 1.0 ml/min, and then oligosaccharides were eluted with 150 mM NaOH. The mobile phase was prepared by diluting carbonate-free HPLC grade 50% (w/w) stock solution in distilled water, filtered with a 0.45 μm membrane, and degassed with compressed nitrogen gas for 30 min before loaded. Glucose mixed with laminarioligosaccharides (10 μg/ml each) (Laminarihexaose, L6; Laminaripentaose, L5; Laminaritetraose, L4; Laminaritriose, L3; Laminaribiose, L2 all from Megazyme) were used as standard substances. Untreated laminarin (80 μg/ml) (Sigma-Aldrich, L9634) was loaded as a blank control. A reaction mixture (100 μl) containing 0.2% laminarin or laminarioligosaccharides and 1.5 μg purified Ebg1-6His protein in 100 mM sodium phosphate (pH 5.0) was incubated at 50 °C for 2 h before loaded in HPLC machine. And a 15 μl aliquot of the end product was diluted with 485 μl of distilled water before ion chromatography (IC) injection.

### Enzymatic activity assay

To assay the enzymatic activity of Ebg1, a 100 μl reaction mixtures containing 0.2% polysaccharides in 100 mM sodium phosphate buffer (pH 5.0) were incubated with 1.5 μg purified Ebg1-6His at 50 °C for 2 h. The glycosyl hydrolase activity of Ebg1 was calculated by the amount of released glucose using a glucose oxidase assay kit (Megazyme)^63^. A 50 μl aliquot of centrifuged supernatant was mixed with 50 μl glucose oxidase standard (GOS) and 200 μl POD overnight in a 96-well bottom clear plate (Costa). Then the absorbance value was measured at 510 nm by a microplate reader (SpectraMax i3x). The gradient glucose standards (50 μl) were used to draw the standard curve. Carbohydrates tested as substrates in this assay were β-1,3-glucan (Sigma-Aldrich, 89862), laminarin (Sigma-Aldrich, L9634), laminarin oligosaccharides (L2-L6) (Megazyme), cellulose (Sigma-Aldrich, S3504), and chitin from shrimp shells (Sigma-Aldrich, C9752). The optimal pH of Ebg1 was evaluated by equilibrating the reaction mixtures with 100 mM sodium acetate (pH 3.5–5.0), sodium phosphate (pH 5.0–7.5), Tris–HCl (pH 7.0–9.0), or sodium phosphate buffer (pH 9.0–12.0) for 12 h at 50 °C^62^. The optimal temperature for Ebg1 was examined by incubating reaction mixtures in 100 mM sodium phosphate (pH 5.0) for 12 h at 4, 10, 20, 30, 40, 50, 60, or 70 °C^62,63^.

### *Agrobacterium tumefaciens* infiltration assay in *N. benthamiana*

The CDS of *EBG1*, *EBG1-N*, *EBG1-C, EBG1*^*E378Q/E476Q*^, and *EF1α* were cloned into the binary vectors pGWB414-3HA or pJL12-3Flag utilizing a pENTR^TM^ Directional TOPO Cloning kit (Invitrogen) and Gateway LR Clonase II enzyme mix (Invitrogen). Constructs were introduced into *A. tumefaciens* strain EHA105 for the agroinfiltration of *N. benthamiana* leaves, as previously reported^38^. PCR confirmed *Agrobacterium* strains were cultured in YEP liquid medium (1% peptone, 1% yeast extract, and 0.5% NaCl, w/v) with suitable antibiotics and re-suspended in infiltration buffer (10 mM MgCl_2_, 10 mM MES, pH 5.6, and 200 μM acetosyringone) to a final OD_600_ of 0.5 for 4 h prior to infiltration^64^. Two days after agroinfiltration, the leaves were punched with a 4 mm-puncher, and then proteins transiently expressed were extracted from 7 leaf disks with 120 μl 2.5 × SDS loading buffer. The proteins were subjected to SDS–PAGE with immunoblot analysis using an anti-HA antibody (Sigma-Aldrich) or an anti-Flag antibody (Sigma-Aldrich, A8592).

For cell death observation, *N. benthamiana* leaves were photographed 5 days post infiltration^36^. The *A. tumefaciens* strain GV3101 carrying the pGR107:2Flag:BAX recombinant construct was used as a positive control for cell death.

For virus-induced gene silencing (VIGS) assays, pTRV1, pTRV2:*GFP*, pTRV2:*PDS*, or pTRV2:*NbBAK1* plasmid constructs were introduced into *A. tumefaciens* strain GV3101. The pTRV2:*GFP*, pTRV2:*PDS*, or pTRV2:*NbBAK1* agrobacteria were mixed with pTRV1 agrobacteria in a 1:1 ratio for co-infiltration. The cultures were infiltrated into the primary leaves of *N. benthamiana* plants at the four-leaf stage. The effectiveness of the VIGS assay was evaluated with pTRV2:*PDS* three weeks after infiltration. The silencing efficiency of *BAK1* was checked by RT-qPCR analysis. *GFP*, *INF1*, and *EBG1* were transiently expressed in the gene-silenced leaves after evaluation, and then leaves were photographed 7 d later.

### ROS burst and MAPK activation assay

The leaves of 4-week-old *N. benthamiana* leaves were punched with a 4 mm diameter puncher for the ROS burst and MAPK assays as described previously^39^. For rice immune responses, 4-week-old rice plants and 2-week-old rice seedlings were used for ROS burst and MAPK assays, respectively. The leaf disks were kept in water overnight before the assays. For ROS burst assay, β-1,3-glucan or glucose were loaded in a final concentration of 20 μg/ml, Ebg1-6His or Ebg1^E378Q/E476Q^-6His proteins were used in a final concentration of 80 μg/ml. The ROS signals were measured in a microplate reader at 450 nm (Spectra Max i3x). For MAPK assay, the activated MAPKs were detected by immunoblotting with the phospho-p42/44 MAPK antibody (CST), and anti-actin antibody (Abclonal) was used as a loading control.

### Quantitative RT-PCR assay

To detect the expression of *EBG1* at different *M. oryzae* infection stages and vegetative growth, barely leaves inoculated with conidial suspensions (8 × 10^5^ spores/ml) of P131 at 0, 18, 24, 36, and 42 hpi were collected and the vegetative hyphae of P131 grown in CM liquid for 24 h were collected for RNA extraction. To detect the expression of *BAK1* in VIGS treated *N. benthamiana* leaves, tobacco leaves with TRV:*GFP* and TRV:*NbBAK1* at 3 weeks after infiltration were collected for RNA extraction. Total RNA was extracted with the KK Fast Plant Total RNA Kit (Zoman Biotechnology, Beijing, China). The first cDNA was synthesized with the HiScript II 1^st^ Strand cDNA Synthesis Kit (Vazyme, Nanjing, China), and Real-Time PCR then performed using 2×RealStar Green Power Mixture with ROXII (GenStar, Beijing, China) using specific primers (Supplementary Table 3). The expression of *EBG1* was normalized to that of *M. oryzae Actin* gene, and the expression of *BAK1* was normalized to *EF1α* of *N. benthamiana*. The experiments were conducted with three biological replicates, and one representative result was shown.

### Yeast two-hybrid (Y2H) assay

The coding sequence (CDS) of *EBG1-∆SP* was cloned into the bait vector pGBKT7 (Clontech) to screen Ebg1-interacting proteins from a cDNA library constructed from with *M. oryzae* infected rice leaf tissues at 18 and 24 hpi. For Y2H validation, the CDS of *EBG1-*∆SP, *EBG1*^*E378Q/E476Q*^*-∆*SP, *EBG1* truncated versions were cloned into pGBKT7, and the CDS of *EF1α* and *EF1α* truncated versions, *MGG_08162* and *MGG_02504* were cloned into pGADT7 (Clontech) (Supplementary Table 3). The resulting bait and prey constructs were confirmed by PCR and then introduced into yeast strain Y2H-Gold as instructed (Clontech, BD library construction & screening kit). The yeast transformants grown on SD-Leu^-^Trp^-^ medium were isolated and assayed for further growth on SD-Leu^-^Trp^-^His^-^ medium. The expression of the Lac Z reporter gene was detected by X-α-gal according to the manufacturer’s instruction.

### Co-immunoprecipitation assay and western blotting

To confirm the interactions of Ebg1 and EF1α in vivo, the genomic sequence of *EBG1* was cloned into pGTN containing a GFP tag under its native promoter, while the genomic sequence of *EF1α* was cloned into pTH3Flag under its native promoter (Supplementary Table 3). The resulting constructs pGTN-*EBG1* and pTH3Flag*-EF1α* were co-transformed into protoplasts of the wild-type strain P131. Transformants were screened on CM plates containing both neomycin (400 μg/ml, Ameresco) and hygromycin B (250 μg/ml, Roche). Total proteins from culture filtrates (CM) of the transformant expressing both Ebg1-GFP and EF1α-3Flag were incubated, respectively, with anti-GFP affinity resins (Chromotek, gta-20) and anti-Flag M2 affinity resins (Sigma-Aldrich, F2426). Proteins bound to resins were eluted after a series of washing steps following the manufacturer’s instruction. The total proteins and elution from resins were detected by western blotting.

For western blotting detecting the presence of proteins, the following antibodes were used, anti-Actin (ABclonal, AC009, 1:2500), anti-Flag (Sigma, A8592, 1:5000), anti-HA (Sigma, H3663, 1:5000), anti-His (Abmart, 10E2, 1:5000), anti-p44/42 (Cell signaling, 9101, 1:2500), anti-GFP (Abclonal, AE012, 1:5000), anti-GST (EASYBIO, BE7012, 1:5000).

### Bioinformatic analyses

*M. oryzae* GH17 family proteins or EF-Tu proteins were identified by a BLAST against NCBI database (http://blast.ncbi.nlm.nih.gov/Blast.cgi) with the amino-acid sequences of *S. cerevisiae* GH17 proteins (Scw4p, Scw10p, Scw11p, and Bgl2p) or *M. oryzae* EF1α. Orthologues of *M. oryzae Ebg1* or *EF1α* were also obtained from NCBI (http://blast.ncbi.nlm.nih.gov/Blast.cgi). Sequences of other mentioned proteins in the article were searched following the NCBI accession numbers in related references. All accession numbers for the predicted protein sequences are provided in Supplementary Figs. 1 or 8.

Protein sequence identity and similarity were determined using EMBOSS Needle (http://www.ebi.ac.uk/Tools/psa/ emboss_needle/). Sequence alignments were done using the MAFFT version7 (https://mafft.cbrc.jp/alignment/server/) and BoxShade (https://embnet.vital-it.ch/software/BOX_form.html). Neighbor-joining phylogenetic trees were calculated in MEGA 6.0, using Bootstrap testing and 1,000 replications. Protein signal peptides were predicted by the SignalP (http://www.cbs.dtu.dk/services/SignalP/). Protein domains were analyzed with SMART (http://smart.embl-heidelberg.de/smart/set_mode.cgi?NORMAL=1). Protein structures of Ebg1 and FgEbg1 were predicted with Alphafold2 (Version 2.2.0, https://github.com/deepmind/alphafold)^65^.

### Statistics and reproducibility

For fungal growth assay, infection assay, DAB staining, and microscrope detection of fluorescence signals from Ebg1-GFP, experiments were repeated at least three times with similar results. For Southern blots and western blots, experiments were repeated at least two times with similar results, and one representative set of results or figures was shown. Image analyses were generated from Image J 1.54d. For all bar plots, data are represented as mean values +/−SD with datapoints shown as dots. Bar plots were drawn using GraphPad Prism 9. Statistical analyses were also performed in GraphPad Prism 9. Statistically significant differences were determined by one-way ANOVA and two-tailed Student’s t-test, and the exact *p* value was provided in the Source data file.

### Reporting summary

Further information on research design is available in the Nature Portfolio Reporting Summary linked to this article.

### Supplementary information

Supplementary information

Description of Supplementary Data

Supplementary Dataset 1-2

Reporting Summary

### Source data

Source Data

## Data Availability

The mass spectrometry proteomics data have been deposited to the ProteomeXchange Consortium (http://proteomecentral.proteomexchange.org) via the iProX partner repository^66,67^ with the dataset identifier PXD024706^68^ and PXD044578. Source data are provided with this paper.

## References

[1] Latge JP (2010). Tasting the fungal cell wall. Cell Microbiol.

[2] Geoghegan I, Steinberg G, Gurr S (2017). The role of the fungal cell wall in the infection of plants. Trends Microbiol..

[3] Langner T (2015). Chitinases are essential for cell separation in *Ustilago maydis*. Eukaryot. Cell.

[4] Mouyna I (2016). GH16 and GH81 family beta-(1,3)-glucanases in *Aspergillus fumigatus* are essential for conidial cell wall morphogenesis. Cell Microbiol.

[5] Mouyna I (2005). Deletion of GEL2 encoding for a beta(1-3)glucanosyltransferase affects morphogenesis and virulence in *Aspergillus fumigatus*. Mol. Microbiol..

[6] Gastebois A, Fontaine T, Latge JP, Mouyna I (2010). beta(1-3)Glucanosyltransferase Gel4p is essential for *Aspergillus fumigatus*. Eukaryot Cell.

[7] Zhao W, Li C, Liang J, Sun S (2014). The *Aspergillus fumigatus* beta-1,3-glucanosyltransferase Gel7 plays a compensatory role in maintaining cell wall integrity under stress conditions. Glycobiology.

[8] Kaku H (2006). Plant cells recognize chitin fragments for defense signaling through a plasma membrane receptor. Proc. Natl Acad. Sci. USA.

[9] Miya A (2007). CERK1, a LysM receptor kinase, is essential for chitin elicitor signaling in Arabidopsis. Proc. Natl Acad. Sci. USA.

[10] Shimizu T (2010). Two LysM receptor molecules, CEBiP and OsCERK1, cooperatively regulate chitin elicitor signaling in rice. Plant J..

[11] Reese TA (2007). Chitin induces accumulation in tissue of innate immune cells associated with allergy. Nature.

[12] Komi DEA, Sharma L, Dela Cruz CS (2018). Chitin and its effects on inflammatory and immune responses. Clin. Rev. Allerg. Immu..

[13] Fesel PH, Zuccaro A (2016). beta-glucan: crucial component of the fungal cell wall and elusive MAMP in plants. Fungal Genet. Biol..

[14] Wanke A (2020). Plant species-specific recognition of long and short beta-1,3-linked glucans is mediated by different receptor systems. Plant J..

[15] Brown GD, Gordon S (2001). Immune recognition—a new receptor for beta-glucans. Nature.

[16] Brown GD, Gordon S (2003). Fungal beta-glucans and mammalian immunity. Immunity.

[17] Mentlak TA (2012). Effector-mediated suppression of chitin-triggered immunity by *Magnaporthe oryzae* is necessary for rice blast disease. Plant Cell.

[18] van den Burg HA, Harrison SJ, Joosten MHAJ, Vervoort J, de Wit PJGM (2006). *Cladosporium fulvum* Avr4 protects fungal cell walls against hydrolysis by plant chitinases accumulating during infection. Mol. Plant Microbe. Interact..

[19] Gao F (2019). Deacetylation of chitin oligomers increases virulence in soil-borne fungal pathogens. Nat. Plants.

[20] Langner T, Gohre V (2016). Fungal chitinases: function, regulation, and potential roles in plant/pathogen interactions. Curr. Genet..

[21] Walton JD (1994). Deconstructing the cell-wall. Plant Physiol.

[22] Hematy K, Cherk C, Somerville S (2009). Host-pathogen warfare at the plant cell wall. Curr. Opin. Plant Biol..

[23] Fuchs Y, Saxena A, Gamble HR, Anderson JD (1989). Ethylene biosynthesis-inducing protein from cellulysin is an endoxylanase. Plant Physiol..

[24] Bailey BA, Dean JFD, Anderson JD (1990). An ethylene biosynthesis-inducing endoxylanase elicits electrolyte leakage and necrosis in *Nicotiana-tabacum Cv xanthi* leaves. Plant Physiol..

[25] Ebbole DJ (2007). *Magnaporthe* as a model for understanding host-pathogen interactions. Annu. Rev. Phytopathol..

[26] Yang C (2019). Binding of the *Magnaporthe oryzae* chitinase MoChia1 by a rice tetratricopeptide repeat protein allows free chitin to trigger immune responses. Plant Cell.

[27] Han YJ (2019). A *Magnaporthe* chitinase interacts with a rice jacalin-related lectin to promote host colonization. Plant Physiol..

[28] Fujikawa T (2009). Dynamics of cell wall components of *Magnaporthe grisea* during infectious structure development. Mol. Microbiol..

[29] Samalova M (2017). The beta-1,3-glucanosyltransferases (Gels) affect the structure of the rice blast fungal cell wall during appressorium-mediated plant infection. Cell Microbiol.

[30] Dean RA (2005). The genome sequence of the rice blast fungus *Magnaporthe grisea*. Nature.

[31] Xue MF (2012). Comparative analysis of the genomes of two field isolates of the rice blast fungus *Magnaporthe oryzae*. PLoS Genet..

[32] Sestak S, Hagen I, Tanner W, Strahl S (2004). Scw10p, a cell-wall glucanase/transglucosidase important for cell-wall stability in *Saccharomyces cerevisiae*. Microbiology.

[33] Kalebina TS (2002). Correct GPI-anchor synthesis is required for the incorporation of endoglucanase/glucanosyltransferase Bg12p into the *Saccharomyces cerevisiae* cell wall. FEMS Microbiol. Lett..

[34] Cappellaro C, Mrsa V, Tanner W (1998). New potential cell wall glucanases of *Saccharomyces cerevisiae* and their involvement in mating. J. Bacteriol..

[35] Mouyna I (1998). A 1,3-beta-glucanosyltransferase isolated from the cell wall of *Aspergillus fumigatus* is a homologue of the yeast Bgl2p. Microbiol-Sgm.

[36] Ma ZC (2015). A *Phytophthora sojae* glycoside hydrolase 12 protein is a major virulence factor during soybean infection and is recognized as a PAMP. Plant Cell.

[37] Heese A (2007). The receptor-like kinase SERK3/BAK1 is a central regulator of innate immunity in plants. Proc. Natl Acad. Sci. USA.

[38] Zhang, Z. & Thomma, B. P. H. J. *Plant-Pathogen Interactions: Methods and Protocols* 2nd edn, Vol. 1127, 173–181 (2014).

[39] Lu X (2009). Uncoupling of sustained MAMP receptor signaling from early outputs in an Arabidopsis endoplasmic reticulum glucosidase II allele. Proc. Natl Acad. Sci. USA.

[40] Chen X (2014). An XA21-associated kinase (OsSERK2) regulates immunity mediated by the XA21 and XA3 immune receptors. Mol. Plant.

[41] Park HS (2011). A subset of OsSERK genes, including OsBAK1, affects normal growth and leaf development of rice. Mol. Cells.

[42] Hu H, Xiong L, Yang Y (2005). Rice SERK1 gene positively regulates somatic embryogenesis of cultured cell and host defense response against fungal infection. Planta.

[43] Yamaguchi T (2000). Differences in the recognition of glucan elicitor signals between rice and soybean: beta-glucan fragments from the rice blast disease fungus *Pyricularia oryzae* that elicit phytoalexin biosynthesis in suspension-cultured rice cells. Plant Cell.

[44] Okmen B, Bachmann D, De Wit PJGM (2019). A conserved GH17 glycosyl hydrolase from plant pathogenic *Dothideomycetes* releases a DAMP causing cell death in tomato. Mol. Plant Pathol..

[45] Dealdana CRV (1991). Nucleotide-sequence of the exo-1,3-beta-glucanase-encoding gene, Exg1, of the yeast *Saccharomyces-cerevisiae*. Gene.

[46] Suzuki K, Yabe T, Maruyama Y, Abe K, Nakajima T (2001). Characterization of recombinant yeast exo-beta-1,3-glucanase (Exg 1p) expressed in *Escherichia coli* cells. Biosci. Biotech. Bioch..

[47] Delrey F, Santos T, Garciaacha I, Nombela C (1979). Synthesis of 1,3-beta-glucanases in *Saccharomyces-cerevisiae* during the mitotic-cycle, mating, and sporulation. J. Bacteriol..

[48] Chambers RS (1993). An exo-beta-(1,3)-glucanase of *Candida-albicans* - purification of the enzyme and molecular-cloning of the gene. J. Gen. Microbiol..

[49] Fu HT (2013). An exo-1,3-beta-glucanase GLU1 contributes to the virulence of the wheat tanspot pathogen *Pyrenophora tritici-repentis*. Fungal Biol..

[50] Rotchanapreeda T (2020). Expression, purification, and characterization of the recombinant exo-1,3-beta-glucanase (Exo1) of the pathogenic oomycete *Pythium insidiosum*. Heliyon.

[51] Keeratijarut A (2015). The immunoreactive exo-1,3-beta-glucanase from the pathogenic oomycete *Pythium insidiosum* is temperature regulated and exhibits glycoside hydrolase activity. PLoS ONE.

[52] Furman-Matarasso N (1999). A point mutation in the ethylene-inducing xylanase elicitor inhibits the beta-1-4-endoxylanase activity but not the elicitation activity. Plant Physiol.

[53] Kunze G (2004). The N terminus of bacterial elongation factor Tu elicits innate immunity in Arabidopsis plants. Plant Cell.

[54] Zipfel C (2006). Perception of the bacterial PAMP EF-Tu by the receptor EFR restricts *Agrobacterium*-mediated transformation. Cell.

[55] Furukawa T, Inagaki H, Takai R, Hirai H, Che FS (2014). Two distinct EF-Tu epitopes induce immune responses in rice and Arabidopsis. Mol. Plant Microbe Interact..

[56] Peng YL, Shishiyama J (1988). Temporal sequence of cytological events in rice leaves infected with *Pyricularia-oryzae*. Can. J. Bot..

[57] Talbot NJ, Ebbole DJ, Hamer JE (1993). Identification and characterization of Mpg1, a gene involved in pathogenicity from the rice blast fungus *Magnaporthe-grisea*. Plant Cell.

[58] Park G, Bruno KS, Staiger CJ, Talbot NJ, Xu JR (2004). Independent genetic mechanisms mediate turgor generation and penetration peg formation during plant infection in the rice blast fungus. Mol. Microbiol..

[59] Chen XL (2014). N-glycosylation of effector proteins by an alpha-1,3-mannosyltransferase is required for the rice blast fungus to evade host innate immunity. Plant Cell.

[60] Kong LA (2012). Different chitin synthase genes are required for various developmental and plant infection processes in the rice blast fungus *Magnaporthe oryzae*. PLoS Pathog..

[61] Jacobs KA (1997). A genetic selection for isolating cDNAs encoding secreted proteins. Gene.

[62] Takahashi M (2010). Characterization of a cellobiohydrolase (MoCel6A) produced by *Magnaporthe oryzae*. Appl. Environ. Microb..

[63] Takahashi M, Konishi T, Takeda T (2011). Biochemical characterization of *Magnaporthe oryzae* beta-glucosidases for efficient beta-glucan hydrolysis. Appl. Microbiol. Biot..

[64] Wang QQ (2011). Transcriptional programming and functional interactions within the *Phytophthora sojae* RXLR effector repertoire. Plant Cell.

[65] Jumper J (2021). Highly accurate protein structure prediction with AlphaFold. Nature.

[66] Ma J (2019). iProX: an integrated proteome resource. Nucleic Acids Res..

[67] Chen T (2021). iProX in 2021: connecting proteomics data sharing with big data. Nucleic Acids Res..

[68] Liu N (2022). Comparative secretome analysis of *Magnaporthe oryzae* identified proteins involved in virulence and cell wall integrity. Genom. Proteom. Bioinf..

