# Supplementary material for: Plant immunity suppression by an exo-β-1,3-glucanase and an elongation factor 1α of the rice blast fungus
Source: Nat Commun. 2023 Sep 7;14:5491. doi: 10.1038/s41467-023-41175-z (PMC10484928; doi:10.1038/s41467-023-41175-z)
Supplement: Supplementary file 1 — Supplementary information [file 41467_2023_41175_MOESM1_ESM.pdf]

Supplementary Information for

Plant immunity suppression by an exo-β-1,3-glucanase and an elongation factor 1α of the rice blast fungus

Hang Liu[1, #], Xunli Lu[1, #], Mengfei Li[1, #], Zhiqin Lun[1], Xia Yan[2], Changfa Yin[1], Guixin Yuan[1], Xingbin Wang[1], Ning Liu[1], Di Liu[1], Mian Wu[1], Ziluolong Luo[1], Yan Zhang[1], Vijai Bhadauria[1], Jun Yang[1], Nicholas J. Talbot[2] and You-Liang Peng[1, ✉]

[1]Ministry of Agriculture and Rural Affairs Key Laboratory for Crop Pest Monitoring and Green Control, China Agricultural University, Beijing 100193, China

[2]The Sainsbury Laboratory, University of East Anglia, Norwich Research Park, Norwich NR4 7UH, UK.

[#]these authors contributed equally.

✉ e-mail: pengyl@cau.edu.cn

Supplementary Figures: 14

Supplementary Tables: 3

Supplementary Figure 1

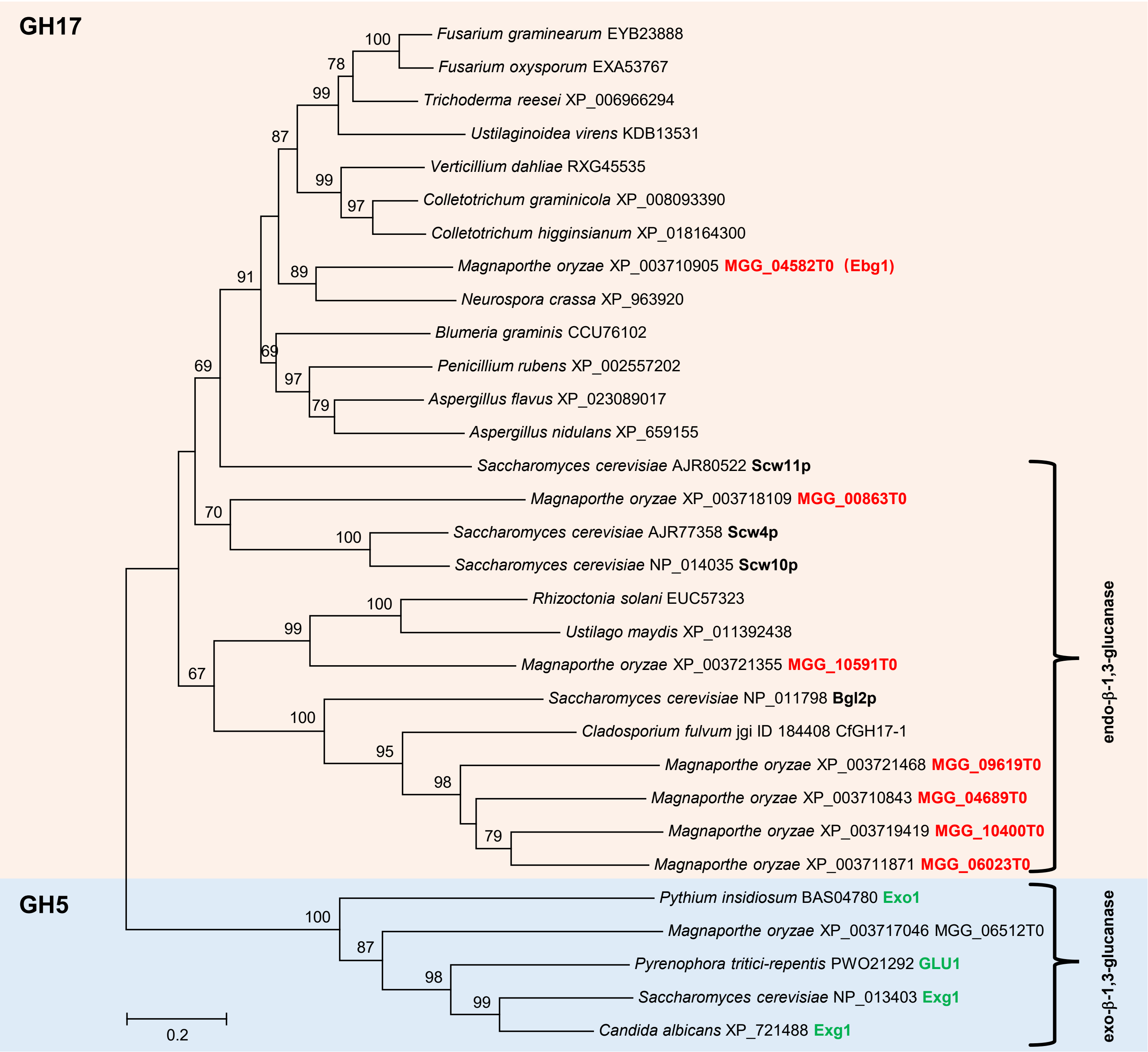

Supplementary Figure 1 | A neighbor-joining tree of *M. oryzae* Ebg1 and its orthologues from other fungi

The tree was constructed in MEGA 6.0. Bootstrap values from 1,000 replications are given at nodes. The scale bar represents 20% weighted sequence divergence. The Exo1, an exo-β-1,3-glucanase of GH5 from the Oomycete Pythium insidiosum, was included in the tree construction(in bold and green). M. oryzae GH17 family proteins are in bold and red. S. cerevisiae GH17 family proteins are in bold and black. Ebg1 has orthologues that have not been functionally characterized in filamentous ascomycetes but lacks orthologues in yeasts and basidiomycetes. It is completely different from the exo-β-1,3-glucanases of GH5 family and also highly divergent from the CfGH17-1, an endo-β-1,3-glucanases of GH17 family from Cladosporium fulvum recently characterized.

Supplementary Figure 2

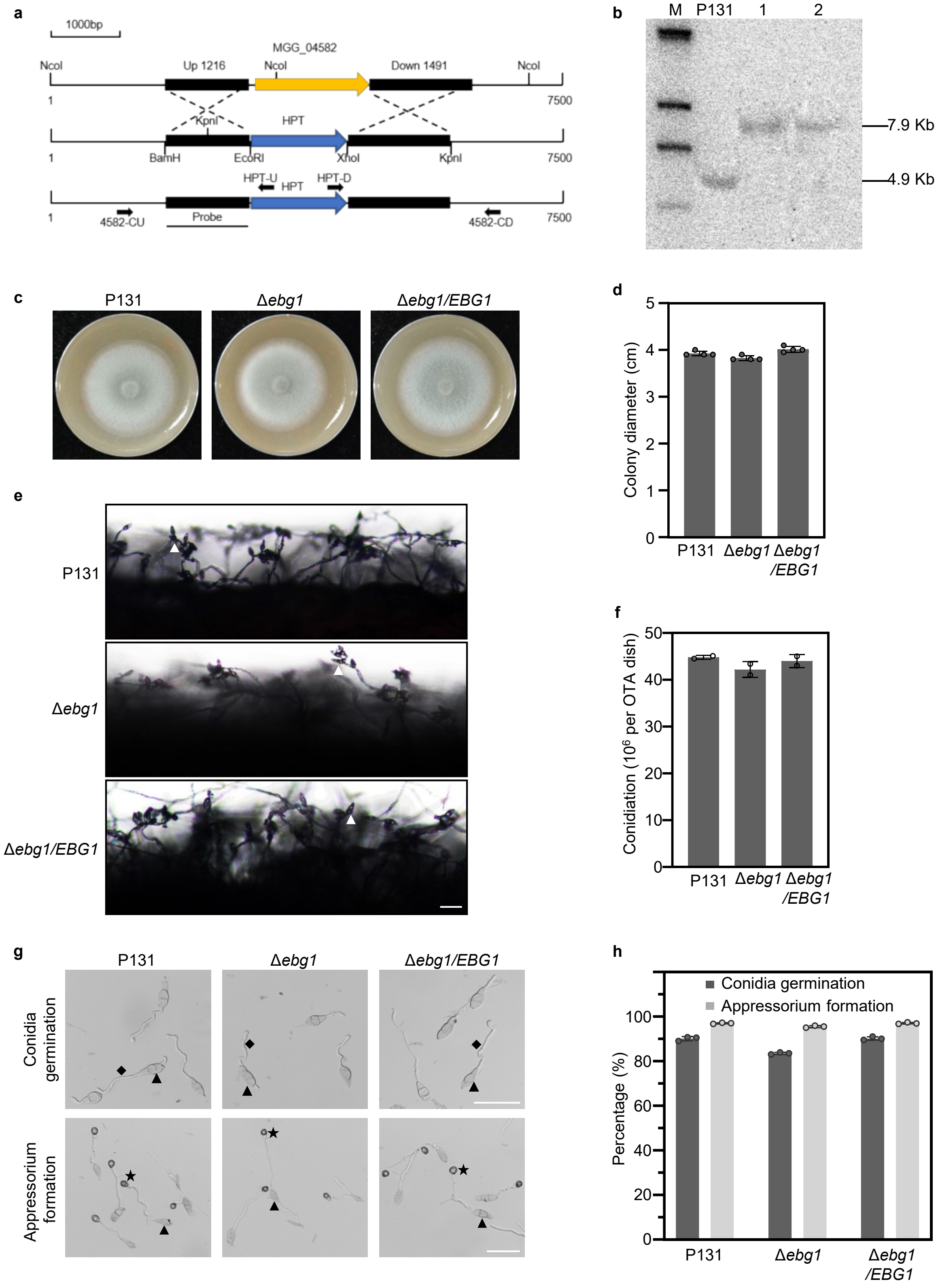

Supplementary Figure 2 | *M. oryzae EBG1* is dispensable for vegetative growth and sporulation

a Schematic diagram of the strategy for EBG1 targeted gene deletion. *Nco*I, *BamH*I, *EcoR*I, *Xho*I and *Kpn*I are enzymes used in vector construction and DNA blot analysis; *HPT*, hygromycin phosphotransferase gene; Probe, 1216 bp upstream *MGG_04582*; HPT-U, HPT-D, 4582-CU, 4582-CD are PCR primers used to screen the *EBG1* deletion mutants.

b, DNA gel blot analysis of the *EBG1* deletion mutants. *Nco*I-digested genomic DNAs were hybridized with the probe mentioned in **a**. M, *Hind*III-digested fragments of *λ*DNA; P131, wild-type strain; 1 and 2, two *EBG1* deletion mutants.

c, d The Δ*ebg1* mutants grow normally on oatmeal–tomato agar plates. Five-day-old oatmeal–tomato agar cultures of P131, Δ*ebg1* mutant and complementation strain Δ*ebg1*/*EBG1* were photographed (**c**), and the colony growth diameters were measured (**d**).

e, f The *EBG1* deletion had no noticeable effect on the conidiation of *M. oryzae*. Conidial formations were induced under white light for 24 h after removing the surface mycelium and then imaged with a light microscope. Small white triangles indicate fresh conidia. Scale bars = 20 μm (**e**). Conidia were collected at 48-hour after induction by washing with 30 ml distilled water per Petri dish (**f**).

g, h Conidial germination and appressorium formation were observed on hydrophobic glass slides and imaged with a light microscope at 2 h and 24 h, respectively. Black triangle, rhombi and star indicated conidia, germ tubes and appressoria, respectively. Scale bars = 20 μm (**g**). The rates of conidia germination and appressorium formation were calculated (**h**).

For all the above statistics, error bars denote standard deviations from three biological replicates. Source data with statistic analysis are provided in a Source Data file.

Supplementary Figure 3

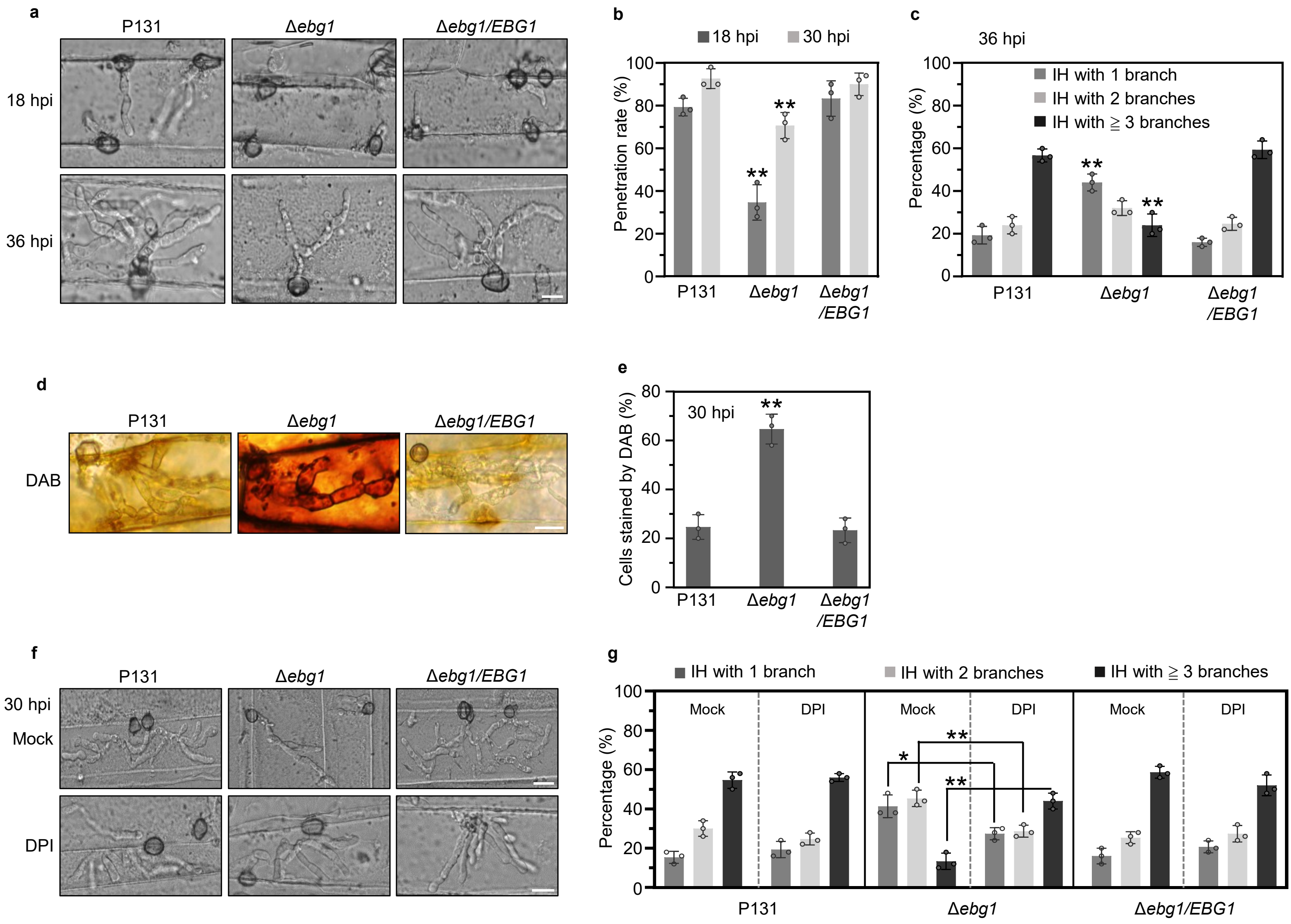

Supplementary Figure 3 | *Magnaporthe oryzae EBG1* is important for invasive growth and suppressing host ROS in barley leaf cells

a, b, c Δ*ebg1* mutants are reduced in invasive growth in barley leaf epidermal cells. Barley leaves were inoculated with conidia suspensions of the P131, Δ*ebg1* and Δ*ebg1*/*EBG1* strains. The hyphal growth was observed and photographed at 18 and 36 hpi with a Nikon 90i microscope (**a**). Scale bars = 20 μm. The Δ*ebg1* mutants are reduced in appressorial penetration rates, which were calculated at 18 and 30 hpi (**b**). The percentage of distinct types of infection hyphae (IH), e. g. one branch, two branches, and three or more branches, were examined at 36 hpi (**c**).

d, e Δ*ebg1* mutants induce ROS production in infected barley cells. Barley leaves drop-inoculated with the conidia suspensions (1×10^5 spores/mL) of the P131, Δ*ebg1* and Δ*ebg1*/*EBG1* strains were stained with DAB at 30 hpi (**d**). Scale bars = 20 μm. The percentages of the DAB-stained infection sites versus total infection sites were calculated (**e**).

f, g Diphenyleneiodonium (DPI), an inhibitor of NADPH oxidase, can partially restore the invasive growth of the Δ*ebg1* mutant. Inoculated barley leaves were treated at 12 h post-inoculation with 0.5 μM DPI dissolved in 1% DMSO and with 1% DMSO as a mock treatment. The growth of infection hyphae was observed and photographed at 30 hpi using a Nikon 90i microscope (**f**). Scale bars = 20 μm. Meanwhile, infection sites with distinct types of infection hyphae were scored to calculate their percentages (**g**).

For all the above statistics, error bars denote standard deviations from three biological replicates. ** and * indicate $p < 0.01$ and $p < 0.05$ significant differences compared with the corresponding WT controls. One-way ANOVA with post-hoc Turkey tests were used in **b**, **c**, **e**, and two-tailed Student's t-test were used in **g**. Source data with statistic analysis are provided in a Source Data file.

Supplementary Figure 4

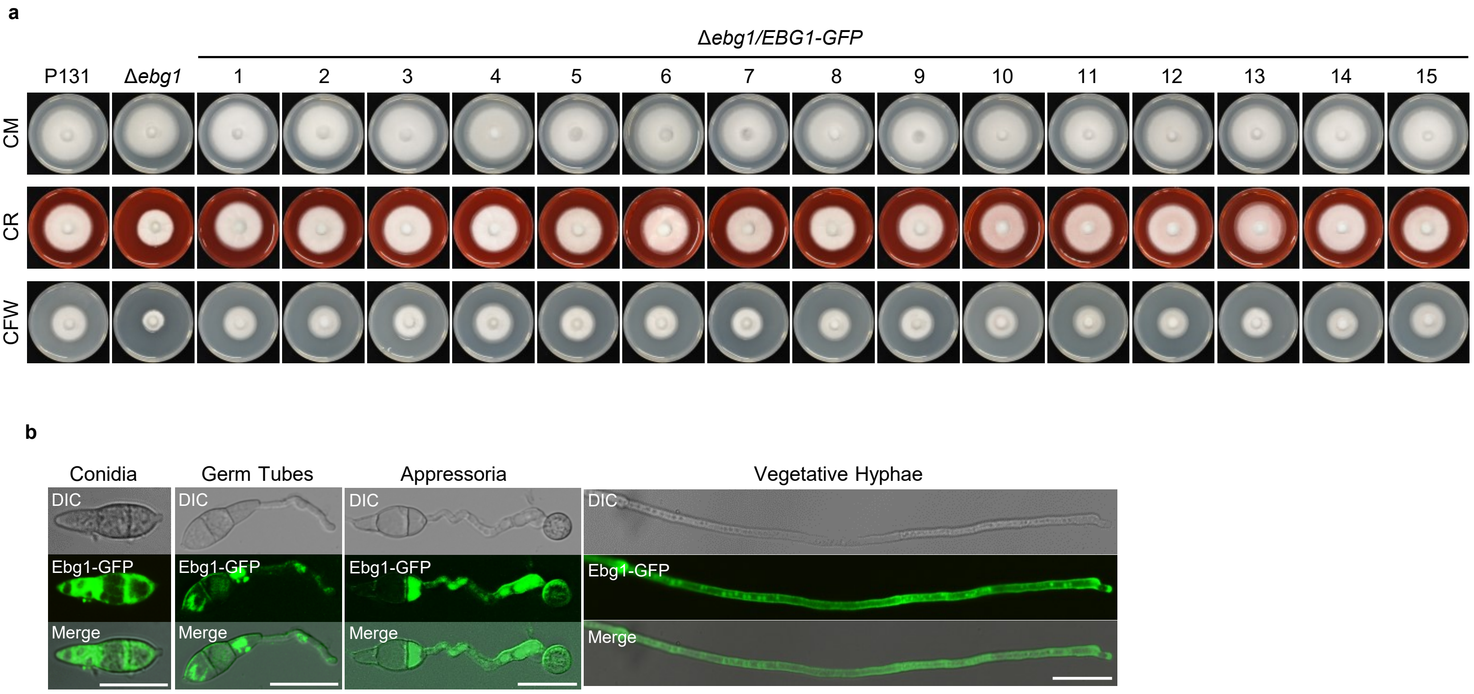

Supplementary Figure 4 | Subcellular distribution of Ebg1-GFP signals in conidia, germ tubes and appressoria of *M. oryzae*

a All the complemented transformants Δ*ebg1*/*EBG1-GFP* recovered wild-type mycelial growth on the complete medium plates supplemented with 200 μg/ml CR or 100 μg/ml CFW at 28°C for 5 days. CR, Congo Red; CFW, Calcofluor white.

b Subcellular localization of Ebg1-GFP in the conidia, germ tube, appressoria and vegetative hyphae of *M. oryzae*. Conidia of a complemented transformant Δ*ebg1*/*EBG1-GFP* expressing Ebg1-GFP were dropped on hydrophobic slides to allow germination and appressorium formation, and Ebg1-GFP fluorescence was unevenly aggregated in conidia, geminated conidia appressoria and vegetative hyphae. Scale bars = 20 μm.

Supplementary Figure 5

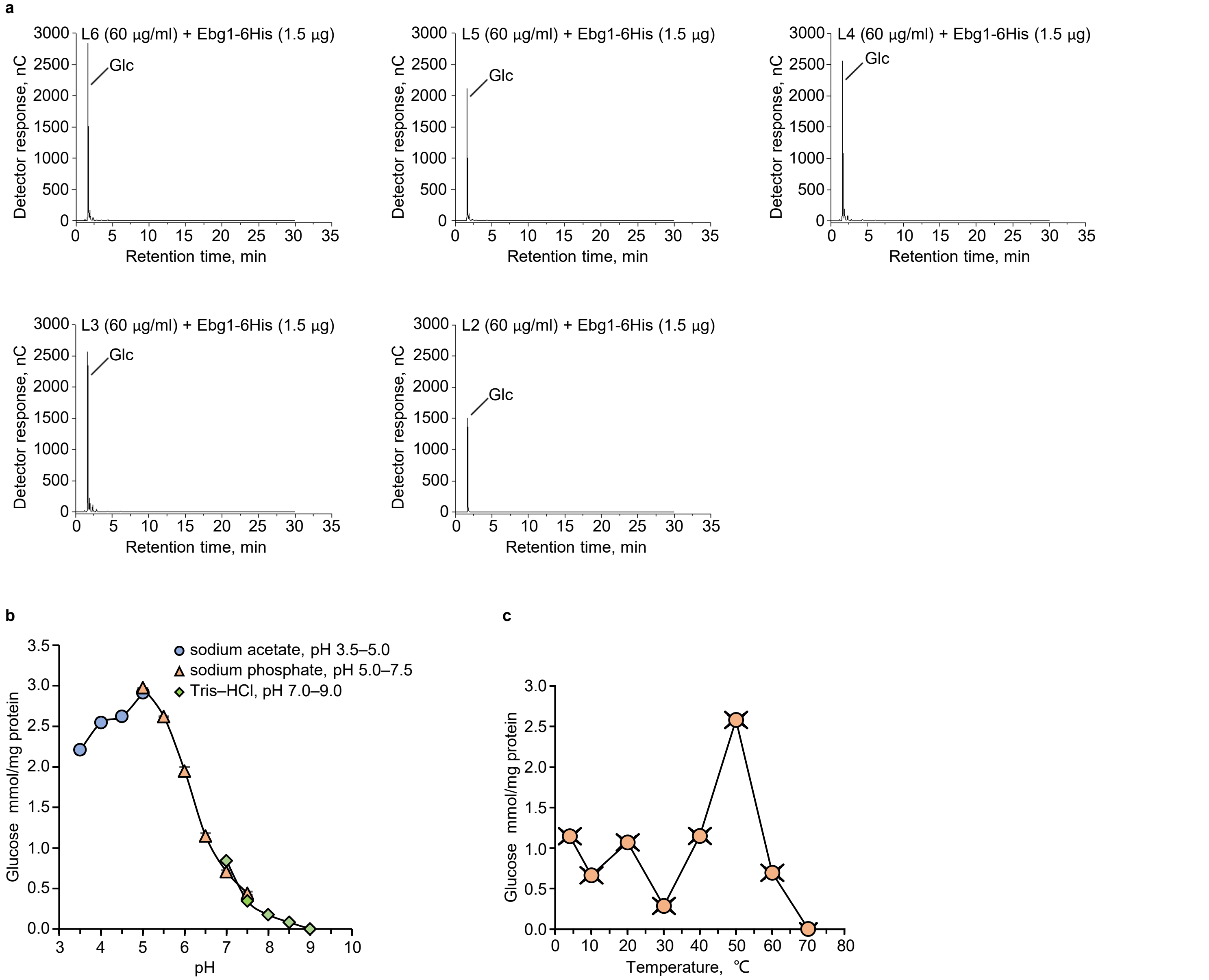

Supplementary Figure 5 | Biochemical characteristics of purified Ebg1

a Purified Ebg1-6His protein hydrolyzed different lengths of laminarin oligosaccharides into glucose. Laminarioligosaccharides with different lengths (L2–L6) treated with purified Ebg1-6His protein were loaded for HPLC analysis. Only glucose was detected from these reaction mixtures. Glc, glucose; L2, Laminaribiose; L3, Laminaritriose; L4, Laminaritetraose; L5, Laminaripentaose; L6, Laminarihexaose.

b Optimal pH for Ebg1 hydrolytic activity. The assay mixtures (100 µl) containing 0.2% Laminarin and 0.3 µg purified Ebg1-6His were incubated at 50°C for 12 h in the presence of 100 mM buffers with different pH: sodium acetate, pH 3.5–5.0; sodium phosphate, pH 5.0–7.5; Tris–HCl, pH 7.0–9.0. A 50 µl aliquot of reaction mixtures was used for enzymatic activity assay.

c Optimal temperature for Ebg1 hydrolytic activity. The assay mixtures (100 µl) containing 0.2% Laminarin and 0.3 µg purified Ebg1-6His were incubated at 4-70°C for 12 h at pH 5.0. A 50 µl aliquot of reaction mixtures was used for enzymatic activity assay. Error bars denote standard deviations from three biological replicates.

Supplementary Figure 6

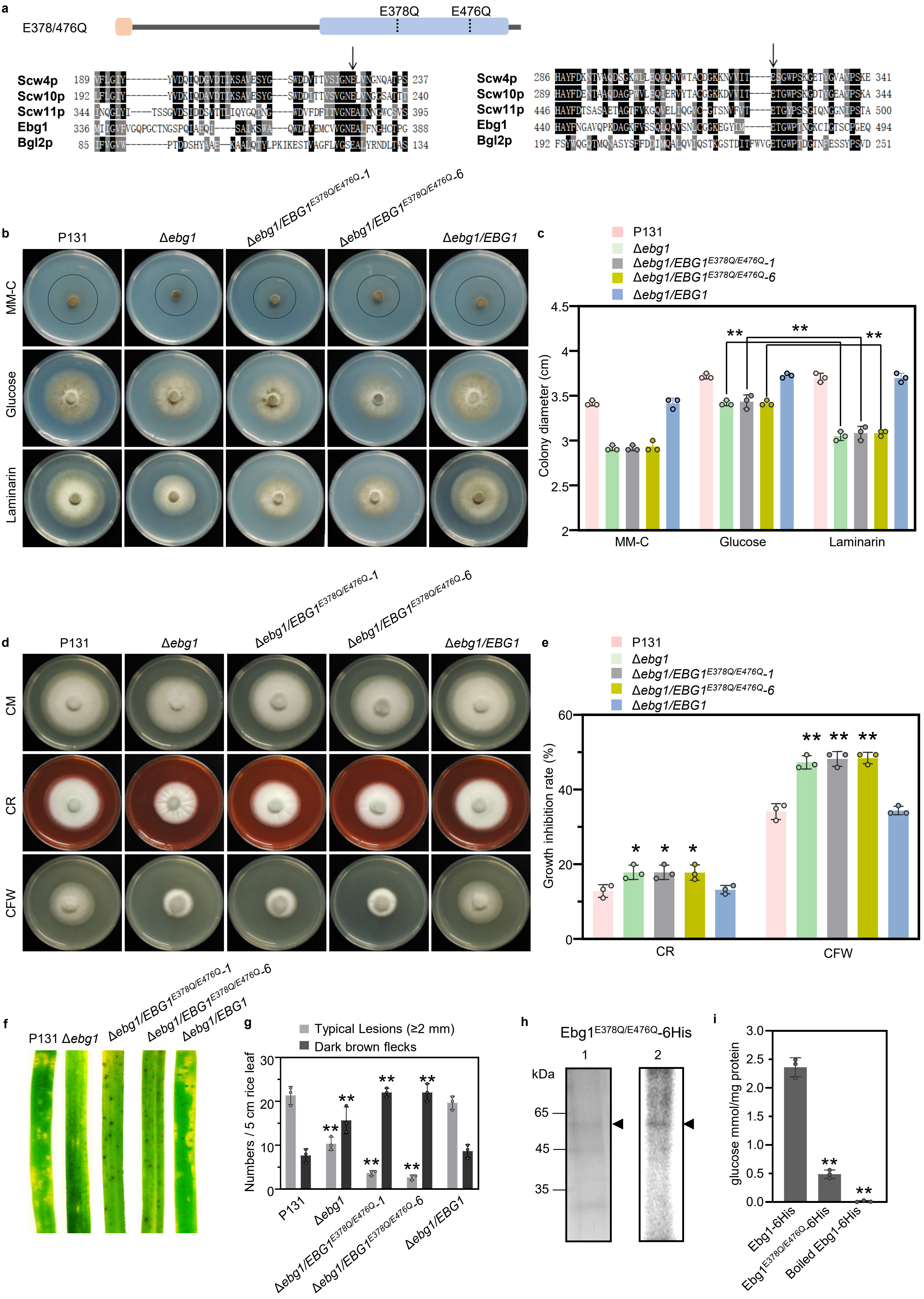

Supplementary Figure 6 | Two conserved glutamic acid residues are essential to the enzymatic functions of Ebg1 in *M. oryzae*

a Schematic diagram of the $EBG1^{E378Q/\ E476Q}$ construct and the alignment of SCW4p, SCW10p, SCW11p, Bgl2p and Ebg1 proteins. E: Glutamic acid; Q: Glutamine. Identical amino acids are shown in black, similar amino acids in grey. Arrows indicate catalytic glutamate residues of GH17 family proteins.

b, c The $\Delta ebg1/EBG1^{E378Q/\ E476Q}$ transformants were similar to the Δ*ebg1* mutants showing limited growth on MM-C plates and unable to be rescued by laminarin. P131, Δ*ebg1* mutant, $\Delta ebg1/EBG1^{E378Q/\ E476Q}$ and Δ*ebg1/EBG1* strains were inoculated at 28°C for 5 days, photographed (**b**) and their colony diameters were measured (**c**).

d, e The $\Delta ebg1/EBG1^{E378Q/\ E476Q}$ transformants were similar to the Δ*ebg1* mutants showing defects in cell wall integrity. P131, Δ*ebg1* mutant, $\Delta ebg1/EBG1^{E378Q/\ E476Q}$ and Δ*ebg1/EBG1* strains were inoculated on CM plates supplemented with Congo Red or Calcofluor white at 28 °C for 5 days, photographed (**d**) and their colony diameters were measured to calculate growth inhibition rates. CR, 200 μg/ml Congo Red; CFW, 100 μg/ml Calcofluor white (**e**).

f, g The $\Delta ebg1/EBG1^{E378Q/\ E476Q}$ transformants were similar to the Δ*ebg1* mutants showing reduced virulence. Rice leaves were sprayed respectively with conidia suspensions (3×10^4 spores/mL) of P131, Δ*ebg1* mutant, $\Delta ebg1/EBG1^{E378Q/\ E476Q}$ and Δ*ebg1/EBG1*. The inoculated leaves were photographed at 5 d post inoculation (**f**), and the numbers of typical lesions and dark brown spots were counted (**g**).

h Purification of the $Ebg1^{E378Q/\ E476Q}$-6His protein from the CM culture filtrates of *M. oryzae*. The crude proteins from liquid CM culture filtrates of a $\Delta ebg1/EBG1^{E378Q/\ E476Q}$*-6His* transformant were purified with ion-exchange chromatography and polyhistidine binding resin before being subjected to SDS–PAGE analysis with CBB staining (panel 1) and immunoblot analysis using the anti-His antibody (panel 2). The solid triangles indicate the correct size for intact $Ebg1^{E378Q/E476Q}$-6His protein at around 55 kDa.

i The $Ebg1^{E378Q/E476Q}$-6His mutant protein largely lose the Ebg1 hydrolytic activity on laminarin. The hydrolytic activities of Ebg1-6His and its mutant $Ebg1^{E378Q/E476Q}$-6His were assayed by detecting the amount of released glucose. Boiled Ebg1-6His was included as a negative control. Error bars denote standard deviation.

For all the above statistics, error bars denote standard deviations from three biological replicates. ** and * indicate $p < 0.01$ and $p < 0.05$ significant differences compared with the corresponding WT controls. One-way ANOVA with post-hoc Turkey tests were used in **e**, **g** and **i**, and Two-tailed Student's t-test were used in **c**.

Supplementary Figure 7

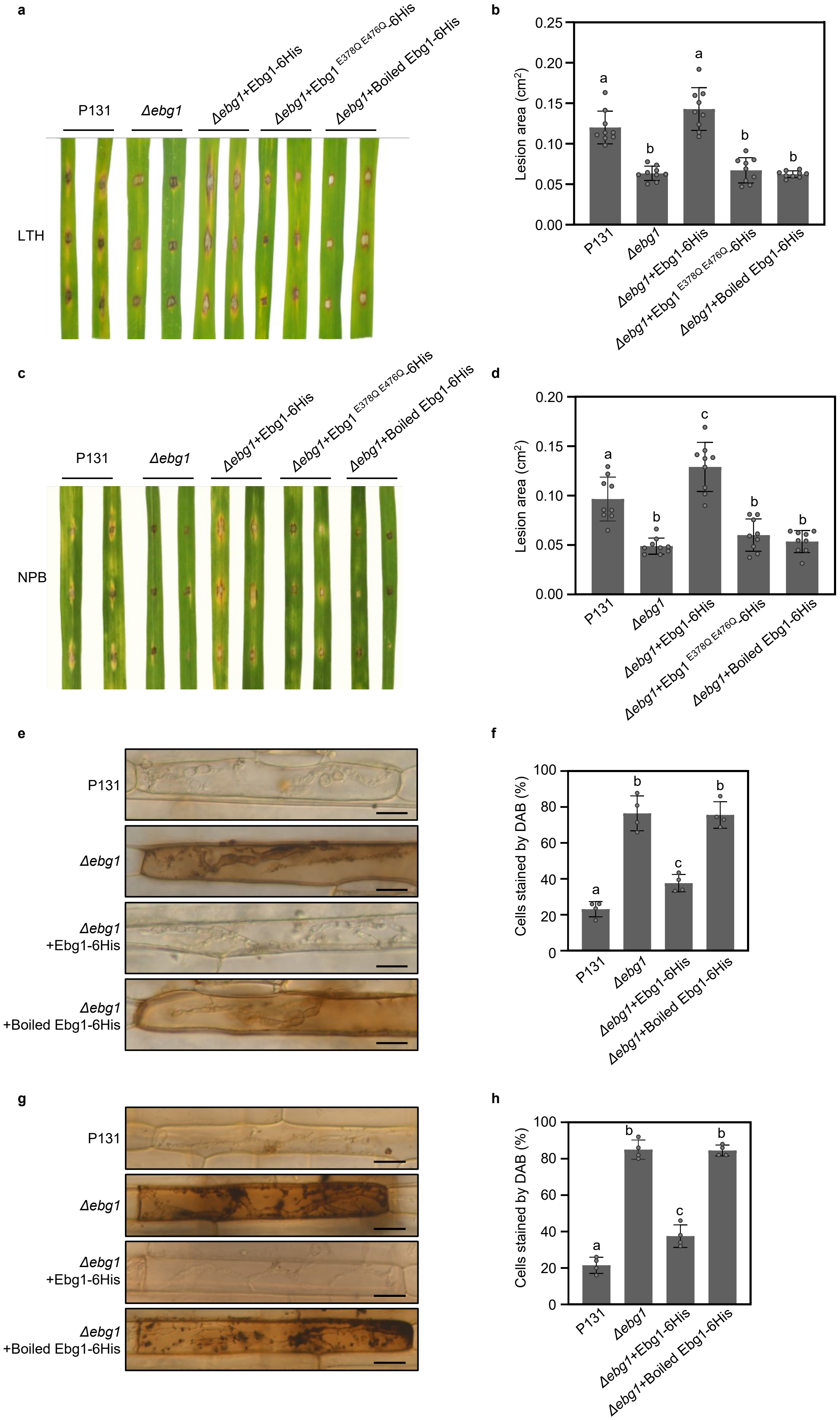

Supplementary Figure 7 | Exogenous application of Ebg1-6His protein rescued the infection defect of the Δ*ebg1* mutant, and suppress the ROS production in plants after Δ*ebg1* infection.

a, b Detached rice leaves of cultiva. LTH were scratched and drop-inoculated with conidia suspensions (1×10^5 spores/mL) of the wild-type P131, *Δebg1*, and *Δebg1* with exogenous application of Ebg1-6His protein (0.2 μg), Ebg1 $^{E378Q\ E476Q}$-6His (0.2 μg) and Boiled Ebg1-6His protein (0.2 μg) and photographed at 5 dpi (**a**). The lesion areas were measured and analyzed (**b**). n = 9.

c, d Detached rice leaves of cultiva. NPB were scratched and drop-inoculated with conidia suspensions (1×10^5 spores/mL) of the wild-type P131, *Δebg1*, and *Δebg1* with exogenous application of Ebg1-6His protein (0.2 μg), Ebg1 $^{E378Q\ E476Q}$-6His (0.2 μg) and Boiled Ebg1-6His protein (0.2 μg) and photographed at 5 dpi (**c**). The lesion areas were measured and analyzed (**d**). n = 9.

e, f Barley leaves drop-inoculated with the conidia suspensions (1×10^5 spores/mL) of P131, Δ*ebg1* and Δ*ebg1* with exogenous application of Ebg1-6His protein (0.2 μg) or Boiled Ebg1-6His protein (0.2 μg) were stained with DAB at 36 hpi (**e**). Scale bars = 20 μm. The percentages of DAB-stained cells versus infected cells were calculated (**f**).

g, h Rice sheath cells inoculated with the conidia suspensions (1×10^5 spores/mL) of P131, Δ*ebg1* and Δ*ebg1* with exogenous application of Ebg1-6His protein (0.2 μg) or Boiled Ebg1-6His protein (0.2 μg) were stained with DAB at 36 hpi (**g**). Scale bars = 20 μm. The percentages of DAB-stained cells versus infected cells were calculated (**h**).

For all the above statistics, error bars denote standard deviations from three biological replicates. Numbers indicate significant differences compared with the corresponding WT controls. One-way ANOVA with post-hoc Turkey tests were used in **b**, **d, f** and **h**.

Supplementary Figure 8

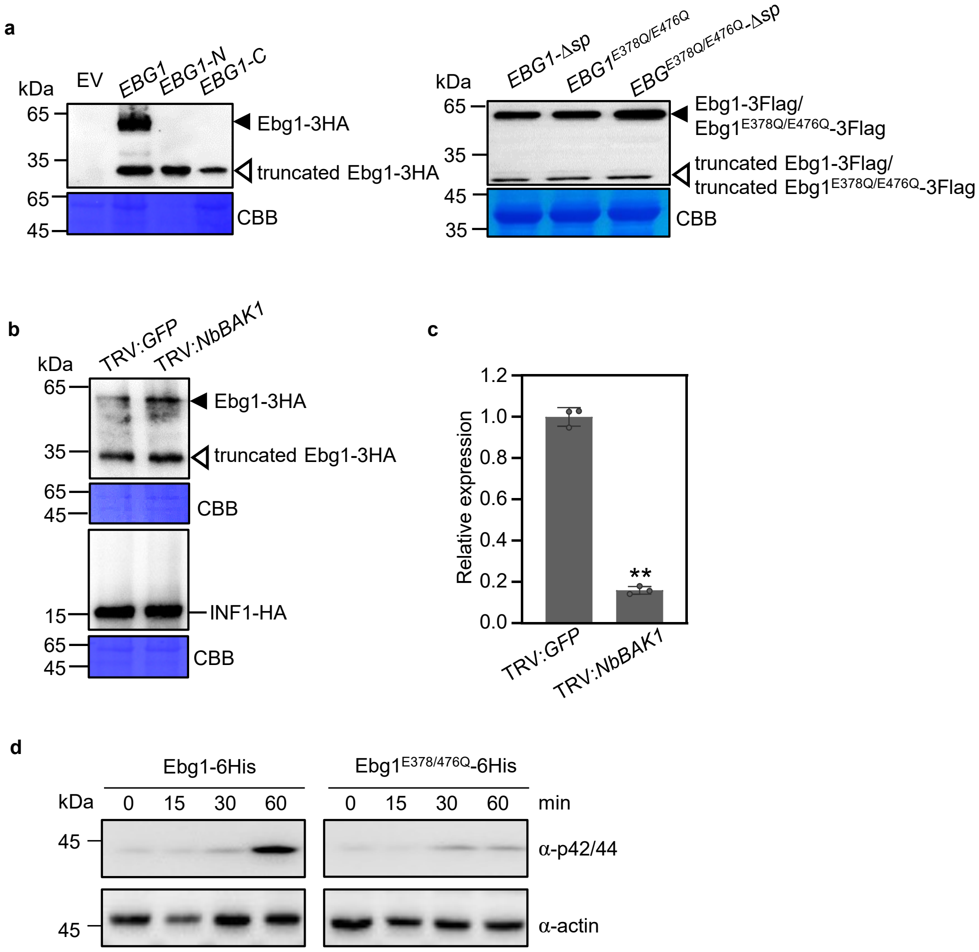

Supplementary Figure 8 | Ebg1 protein induces cell death in *N. benthamiana* and immune responses in rice leaves

a The expression of *BAX*, *EBG1*, *EBG1-N*, *EBG1-C*, and *EBG1*$^{E378Q/E476Q}$ in *N. benthamiana* was validated by immunoblotting using anti-Flag antibody or anti-HA antibody. CBB, Coomassie brilliant blue staining was used to ensure equal sample loading. The black solid triangles indicate the correct size for Ebg1-3HA or Ebg1-3Flag proteins, and the open triangles indicate the truncated proteins.

b The expression of *INF1* and *EBG1* in BAK1-silenced *N. benthamiana* was validated by immunoblot analysis using the anti-HA antibody. CBB, Coomassie brilliant blue staining was used to ensure equal sample loading. Intact and truncated versions of Ebg1-3HA were labeled.

c The expression of *BAK1* was reduced in TRV:*NbBAK1* silencing line of *N. benthamiana.* The relative expression level of *BAK1* was normalized to plant *EF1α* by RT-qPCR analysis, and the value in TRV:GFP was set as 1. Error bars denote standard deviations from three biological replicates. ** indicates $p < 0.01$ significant difference compared with the corresponding control using two-tailed Student's t-test.

d Purified Ebg1-6His and Ebg1$^{E378Q/E476Q}$-6His both induce MAPK activation in rice cultivar ZH11. Two-week-old rice seedlings were incubated with 100 μg/ml for the indicated time. Activated MAPKs were detected by immunoblotting with the phospho-p42/44 MAPK antibody. The anti-actin blot was used as a loading control. The experiment was repeated two times.

Supplementary Figure 9

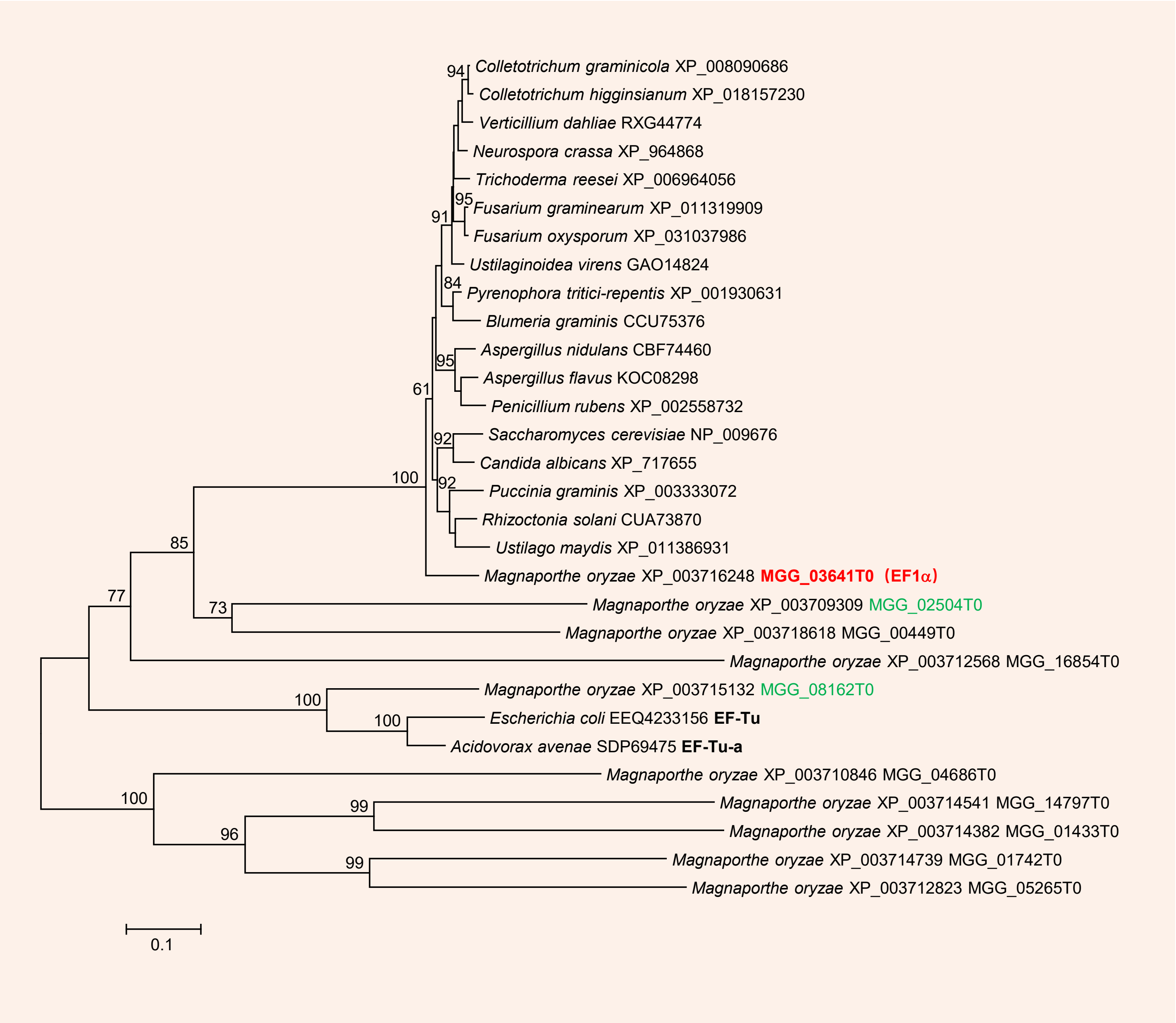

Supplementary Figure 9 | A Neighbor-joining tree of *M. oryzae* EF1α and its orthologues and related proteins from fungi, *Escherichia coli* and *Acidovorax avenae*

The tree was constructed in MEGA 6.0. Bootstrap values from 1,000 replications are given at nodes. The scale bar represents 10% weighted sequence divergence. *M. oryzae* EF1α is in bold and red. *E. coli* EF-Tu and *A. avenae* EF-Tu-a are in bold and black. Two EF-Tu-like proteins of *M. oryzae* related to EF1α are in bold and green.

Supplementary Figure 10

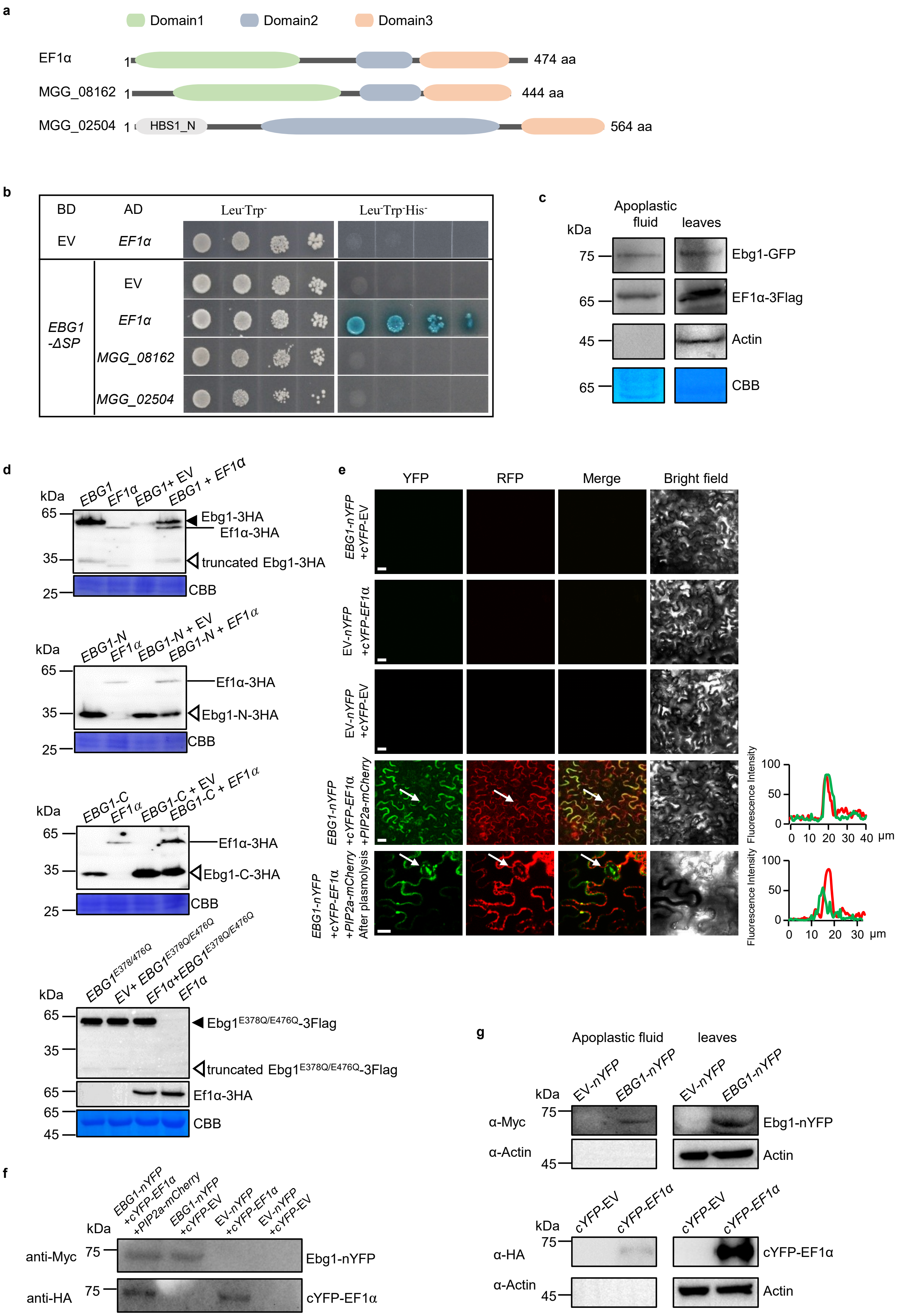

Supplementary Figure 10 | Ebg1 interacts with EF1α, but not two EF1α -related proteins, in *M. oryzae*

a Schematic diagram of EF1α and two homologues, MGG_08162 and MGG_02504 in *M. oryzae*. All the domains are indicated by various color boxes. Domain1, GTP binding domain; Domain2 and Domain3, beta-barrel structure.

b The yeast two-hybrid assay showing the interaction of Ebg1 with EF1α but not with its homologues. AD-*EF1α*, AD-*MGG_08162*, AD-*MGG_02504* and BD-*EBG1-ΔSP* plasmids were respectively co-transformed into yeast cells and screened on synthetic dextrose media lacking leucine and tryptophan (SD-Leu-Trp-). Single colonies were diluted onto SD-Leu-Trp- and SD-Leu-Trp-His- (synthetic dextrose media lacking leucine, tryptophan, and histidine) to observe the yeast cell growth. Yeast cells on SD-Leu-Trp-His- were stained by x-a-gal. Yeast co-transformed with AD (empty vector) and BD-*EBG1-ΔSP*, besides BD (empty vector) and AD-*EF1α*, both served as a negative control.

c Both Ebg1-GFP and EF1α-3Flag were detected from the apoplastic fluid of barley leave. Barley seedlings were infected by a *M. oryzae* transformants of *EBG1-GFP* and *EF1α-3Flag* strain, and then the protein extraction from leave fluid and leaves were separately collected. Immunoblots were performed using anti-GFP antibody and anti-Flag antibody. The anti-Actin antibody was used to detect the plant endogenous actin as a loading control. CBB, Coomassie brilliant blue staining indicates the total extracted protein from each sample.

d Immunoblots with anti-HA or anti-Flag antibody validated the expression of Ebg1-3HA, Ebg1-N-3HA, Ebg1-C-3HA and Ebg1$^{E378Q/E476Q}$-3Flag in *N. benthamiana* co-infiltrated with or without EF1α-HA. CBB, Coomassie brilliant blue staining was used to ensure equal sample loading. The black solid triangles indicate the correct size for Ebg1-3HA or Ebg1$^{E378Q/E476Q}$-3Flag proteins, and the open triangles indicate the truncated proteins.

e BiFC assays showing the interaction of Ebg1 with EF1α in *N. benthamiana*. *N. benthamiana* leaves were co-infiltrated with agrobacteria with *35S::EBG1-nYFP*, *35S::cYFP-EF1α* and *35S::PIP2a-mCherry* (a plasma membrane marker). Plasmolysis treatment with 10 mM NaCl showed that the localization of Ebg1-nYFP and cYFP-EF1α is separated from PIP2a-mCherry. Scale bars = 20 µm. The fluorescence curves at the right bottom were obtained following the direction of white arrows.

f Immunoblotting showing expression of proteins described in **e**.

g Ebg1 and EF1α can enter into the apoplast of *N. benthamiana*. Apoplast proteins were extracted from *N. benthamiana* leaves co-expressing *EBG1-nYFP* and *cYFP-EF1α*. Proteins from apoplast and *N. benthamiana* leaves were detected using anti-Myc antibody and anti-HA antibody. The anti-Actin antibody was used to detect the plant endogenous actin as the loading control.

Supplementary Figure 11

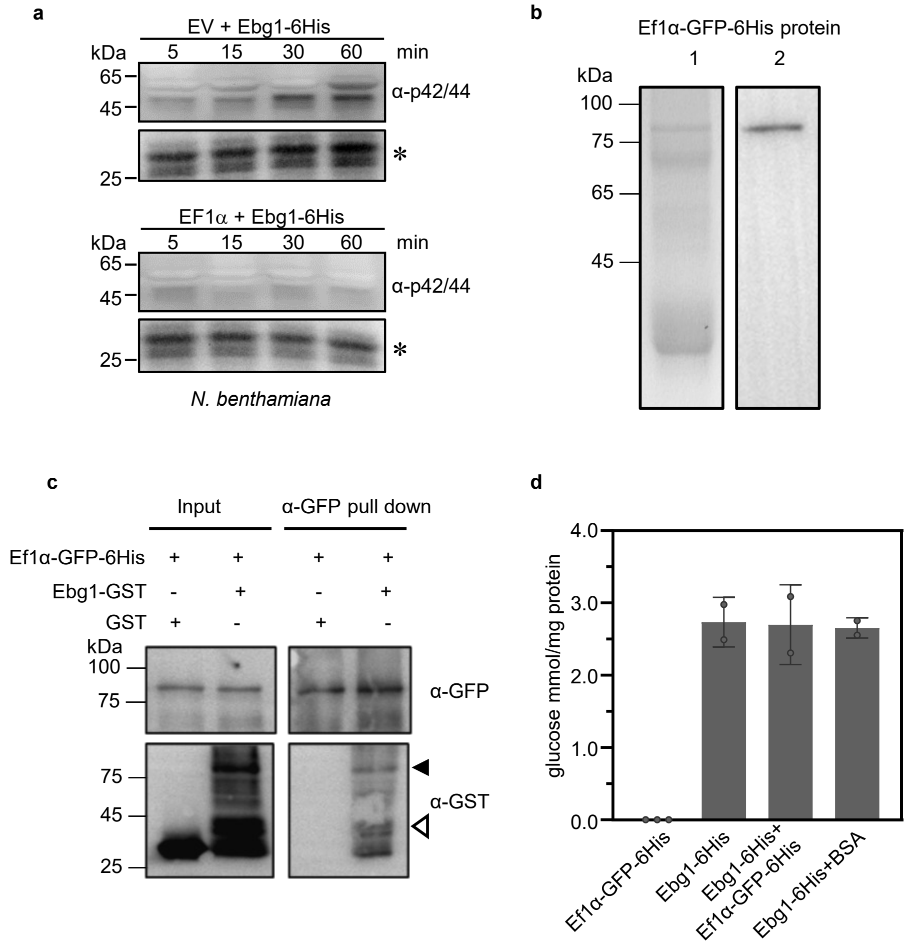

Supplementary Figure 11 | Ef1α inhibits the Ebg1-induced cell death in tobacco, but not the enzyme activity of Ebg1

a Expression of *EF1α* prevents MAPK activation triggered by Ebg1-6His protein in *N. benthamiana*. *N. benthamiana* leaves were first infiltrated with agrobacteria harboring *EF1α* or with empty vector (EV) for 1 day, and then incubated with purified Ebg1-6His protein (2µg/ml). Activated MAPKs were detected by immunoblotting with the phospho-p42/44 MAPK antibody at the indicated time points. *, unspecific bands indicating equal sample loading. The assays were repeated three times.

b Purification of Ef1α -GFP-6His protein from the yeast strain. Purified Ef1α-GFP-6His protein was subjected to SDS–PAGE analysis with CBB staining (lane 1) and immunoblot analysis using the anti-His antibody (lane 2).

c Pull-down assay confirming that the Ef1α-GFP-6His protein interacts with Ebg1-GST protein *in vitro*. Co-incubated Ef1α-GFP-6His and Ebg1-GST proteins were subjected to GFP pull-down analysis. Total proteins and pull-down elution were detected by anti-GFP antibody or anti-GST antibody. Co-incubated Ef1α-GFP-6His and GST proteins were used as a negative control. The black solid and open triangles point to the signals for intact Ebg1-GST protein and the truncated Ebg1-GST protein, respectively.

d The purified EF1α-GFP-6His protein does not inhibit the enzyme activity of Ebg1-6His. The hydrolytic activities of Ebg1-6His on laminarin were assayed by detecting the amount of released glucose. EF1α-GFP-6His protein itself does not have the enzyme activity, thus is used as a negative control. Error bars denote standard deviations from two biological replicates.

Supplementary Figure 12

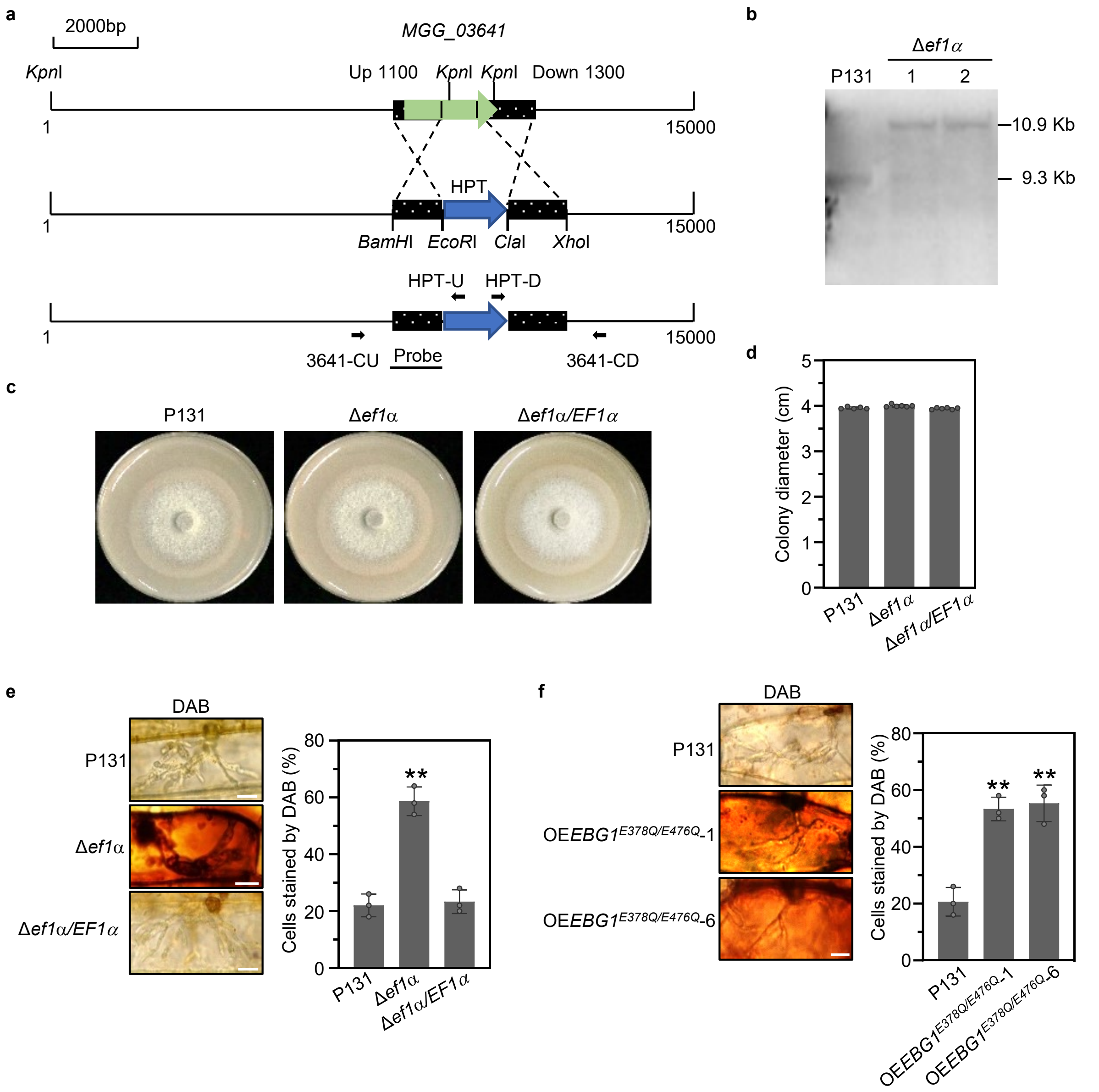

Supplementary Figure 12 | *M. oryzae EF1α* is dispensable for vegetative growth and development

a Schematic diagram of the *EF1α* targeted gene deletion strategy. *BamH*I, *EcoR*I, *Cla*I, *Xho*I and *Kpn*I were enzymes used in vector construction and DNA gel blot analysis; *HPT*, hygromycin phosphotransferase gene; Probe, 1100 bp upstream *MGG_03641*; HPT-U, HPT-D, 3641-CU and 3641-CD are PCR primers used to screen *EF1a* deletion mutants.

b DNA gel blot analysis of the *EF1α* deletion mutants. *Kpn*I-digested genomic DNAs were hybridized with the probe indicated in **a**. P131, wild-type strain; 1 and 2, two *EF1a* deletion mutants.

c, d The *Δef1α* mutant grows normal on oatmeal–tomato agar plates. Five-day-old cultures of P131, a Δ*ef1α* mutant and a complemented transformant Δ*ef1α*/*EF1α* were photographed (**c**), and the colony growth diameters were measured (d).

e The Δ*ef1a* mutants fail to prevent ROS production in barley leaf epidermal cells. Barley leaves drop-inoculated with conidia suspensions (1×10^5 spores/mL) of the P131, Δ*ef1a* and Δ*ef1a*/*EF1a* strains were stained with DAB at 30 hpi, and the percentage of infection sites with DAB stained cells was calculated. Scale bars = 20 μm.

f The $EBG1^{E378Q/E476Q}$ mutants induced ROS production. Barley leaves drop-inoculated with conidial suspensions (1×10^5 spores/ml) of P131 or $EBG1^{E378Q/E476Q}$ transformants were stained with DAB at 30 hpi, and the percentages of the DAB-stained infection sites were calculated. Scale bars = 20 μm.

For all the above statistics, error bars denote standard deviations from three biological replicates. ** indicates $p < 0.01$ significant differences compared with the corresponding WT controls. One-way ANOVA with post-hoc Turkey tests were used in **d**, **e** and **f**,

Supplementary Figure 13

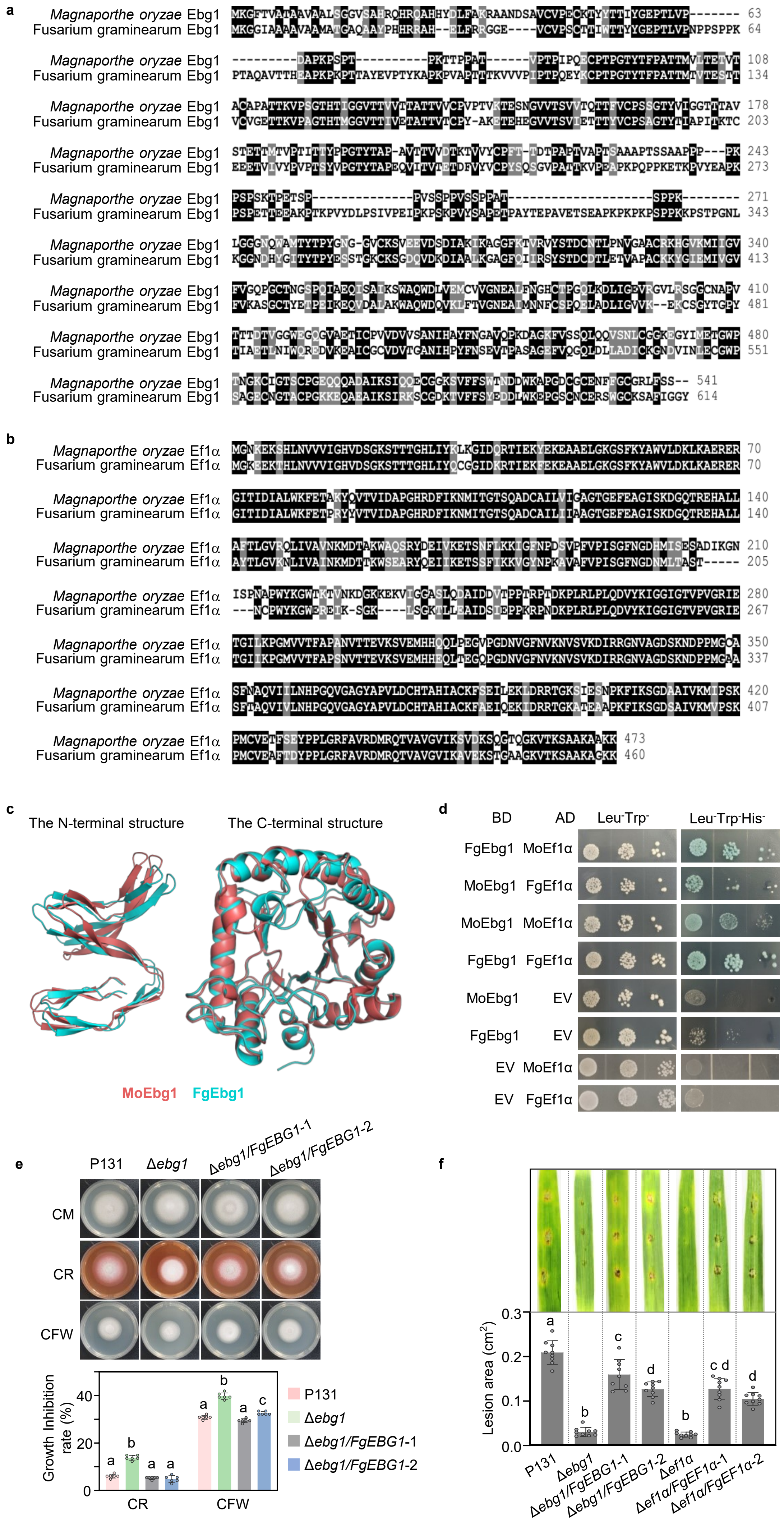

Supplementary Figure 13 | Fungal orthologous Ebg1 and EF1α may be functionally conserved

a, b Amino acid sequence alignments showing that Ebg1 of *M. oryzae* (MoEbg1) is divergent from FgEbg of *F. graminearum* and that Ef1α of *M. oryzae* (MoEf1α) is highly similar to FgEf1α of *F. graminearum*, respectively. The alignment was performed with MAFFT version 7 and BoxShade.

c MoEbg1 (in pink) is highly similar to FgEbg1 (in light blue) in predicted structure. The structures of MoEbg1 and FgEbg1 were predicted with Alphafold2.

d Yeast two-hybrid assays showing that FgEbg1 can interact with FgEf1α and with MoEf1α, and that MoEbg1 can also interact with FgEf1α in addition to MoEf1α in yeast cells.

e FgEbg1 could rescue the cell wall integrity deficiency of *M. oryzae* Δ*ebg1* mutants. The wild-type P131, *Δebg1*, and two *Δebg1/FgEBG1* strains were cultured on the complete medium (CM) plates supplemented with 200 μg/ml CR or 100 μg/ml CFW at 28°C for 5 days (upper panel), and their growth inhibition rates were calculated (lower panel). CR, Congo Red; CFW, Calcofluor white.

f FgEbg1 and FgEf1α could largely rescue the reduced virulence phenotypes of corresponding *M. oryzae* Δ*ebg1* and Δ*ef1α* mutants. Detached barley leaves were drop-inoculated with conidia suspensions (5×10^4 spores/mL) of the wild-type P131, *Δebg1*, and two *Δebg1/FgEBG1* strains, *Δef1α*, and two *Δef1α/FgEF1α* strains, and photographed at 5 dpi (upper panel). The lesion areas were measured and analyzed (lower panel).

For all the above statistics, error bars denote standard deviations from three biological replicates. Numbers indicate significant differences between the samples using one-way ANOVA with post-hoc Turkey in **e** and **f**.

Supplementary Figure 14

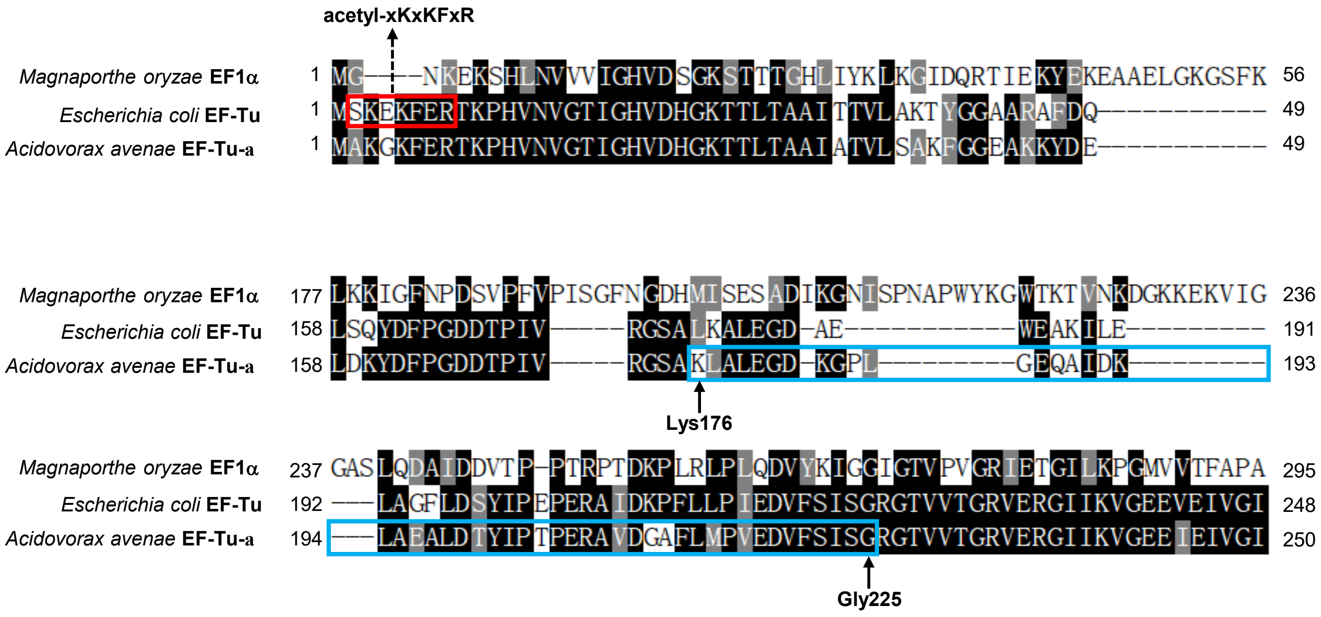

Supplementary Figure 14 | Protein sequence alignments of *M. oryzae* EF1α with *E. coli* EF-Tu and *A. avenae* EF-Tu-a

Protein sequence alignments were conducted by MAFFT version 7 and BoxShade. The elicitor activity motif of *E. coli* EF-Tu is acetyl-xKxKFxR indicated in the red frame. The elicitor activity motif of *A. avenae* EF-Tu-a is EFa50 from Lys176 to Gly225 indicated in the blue frame.

Supplementary Table 1: Candidate Ebg1-interacting proteins from yeast-two-hybrid Screening

No. of Clones	Gene	Putative function	Species
5	MGG_10568	cycloartenol-C-24-methyltransferase 1-like isoform	
25	MGG_17265	glycogen/starch/alpha-glucan phosphorylase	*Magnaporthe oryzae*
**36**	**MGG_03641**	**elongation factor 1 alpha**	
50	MGG_07584	putative serine peptidase	
4	LOC_Os04g43130.1	transcriptional corepressor LEUNIG	*Oryza sativa*
21	LOC_Os05g02070.2	metallothionein-like protein 2C	
29	LOC_Os04g02890.1	UPF0548 protein	
30	LOC_Os03g01730.1	UPF0548 protein	
51	LOC_Os10g39620.1	ubiquitin family domain containing protein	

Supplementary Table 2: List of strains used in this study

Strains	Genotype description	Reference
P131	Wild type	Peng, Y.-L. & Shishiyama, J., 1988
Δ*ebg1*	*EBG1* deletion mutant of P131	This study
Δ*ebg1/EBG1*	EBG1-GFP complementary transformant of Δ*ebg1*	This study
Δ*ebg1/EBG1-6His*	EBG1-6His complementary transformant of Δ*ebg1*	This study
Δ*ebg1/EBG1*$^{E378Q\ E476Q}$	EBG1$^{E378Q\ E476Q}$ complementary transformant of Δ*ebg1*	This study
Δ*ebg1/EBG1*$^{E378Q\ E476Q}$*-6His*	EBG1$^{E378Q\ E476Q}$ -6His complementary transformant of Δ*ebg1*	This study
P131/OE*EBG1*$^{E378Q\ E476Q}$	EBG1$^{E378Q\ E476Q}$ overexpression transformant of P131	This study
P131/*GFP*	GFP transformant of P131	This study
Δ*ef1α*	*EF1α* deletion mutant of P131	This study
Δ*ef1α/EF1α*	EF1α-GFP complementary transformant of Δ*ef1α*	This study
P131/*EBG1-GFP* + *EF1α–3Flag*	EBG1-GFP and EF1α-3Flag co-transformant of P131	This study
P131/*SLP1-GFP* + *EF1α–3Flag*	SLP1-GFP and EF1α-3Flag co-transformant of P131	This study
EHA105/*EV*	Empty vector (pGWB414:3HA) transformant of *A. tumefaciens* strain EHA105, tobacco cell death negative control	This study
EHA105/*EBG1-3HA*	EBG1 expression transformant of EHA105	This study
EHA105/*EBG1-N-3HA*	EBG1-N expression transformant of EHA105	This study
EHA105/*EBG1-C-3HA*	EBG1-C expression transformant of EHA105	This study
EHA105/*EF1α-3HA*	EF1α expression transformant of EHA105	This study
EHA105/*GFP*	GFP expression transformant of EHA105	This study
EHA105/*INF1-3HA*	INF1 expression transformant of EHA105	This study
EHA105/*EV-nYFP*	EV-nYFP transformant of EHA105, BiFc assay negative control	This study
EHA105/*cYFP-EV*	cYFP-EV transformant of EHA105, BiFc assay negative control	This study
EHA105/*EBG1-nYFP*	EBG1-nYFP expression transformant of EHA105	This study
EHA105/*cYFP-EF1α*	cYFP-EF1α expression transformant of EHA105	This study
EHA105/*PIP2a-mCherry*	PIP2a-mCherry transformant of EHA105, a plasma membrane marker	This study
GV3101/*EBG1-ΔSP-3Flag*	*EBG1-ΔSP* transformant of *A. tumefaciens* strain GV3101	This study
GV3101/*EBG1*$^{E348Q/476Q}$*-3Flag*	*EBG1*$^{E348Q/476Q}$ transformant of *A. tumefaciens* strain GV3101	This study
GV3101/ *EBG1*$^{E348Q/476Q}$*-ΔSP-3Flag*	*EBG1* expression transformant of *A. tumefaciens* strain GV3101	This study
GV3101/*EF1α -3HA*	*EF1α* expression transformant of *A. tumefaciens* strain GV3101	This study
GV3101/*BAX*	BAX expression transformant of *A. tumefaciens* strain GV3101, tobacco cell death positive control	This study

GV3101/TRV2:*GFP*	*GFP* silencing transformant of GV3101, tobacco gene silencing negative control	This study
GV3101/TRV2:*NbBAK1*	*NbBAK1* silencing transformant of GV3101	This study
GV3101/TRV2:*PDS*	*PDS* silencing transformant of GV3101	This study
YTK12/*EBG1*	EBG1 expression transformant of yeast strain YTK12	This study
YTK12/*EBG1-ΔSP*	EBG1-ΔSP expression transformant of yeast strain YTK12	This study
YTK12/*Avr1b*	Avr1b expression transformant of yeast strain YTK12, yeast secretion positive control	This study
YTK12/*Mg87*	Mg87 expression transformant of yeast strain YTK12, yeast secretion negative control	This study
GS115/*EBG1-GFP-6His*	EBG1-GFP-6His expression transformant of *P.pastoris* strain GS115	This study
GS115/*EBG1*$^{E378Q\ E476Q}$ -*GFP-6His*	EBG1$^{E378Q\ E476Q}$-GFP-6His expression transformant of *P.pastoris* strain GS115	This study
GS115/*EF1α -GFP-6His*	*EF1α* -GFP-6His expression transformant of *P.pastoris* strain GS115	This study
BL21/*GST*	Empty vector (pGEX:GST) transformant of *E.coli* strain BL21 (DE3), pull down negative control	This study
BL21/*EBG1-GST*	EBG1-GST expression transformant of BL21 (DE3)	This study

Supplementary Table 3: Primer sequences used in this study.

Primer (5'--3')	Sequence (5'--3')	Description
actin qPCR F	TCCTTGCCCCACGCCATTGC	Expression measurement
actin qPCR R	CTCCAAGCTGGAGCTCT	Expression measurement
04582 qPCR F	TCCGTGTTTACTCGACCGAC	Expression measurement
04582 qPCR R	TCTCAACGAGGTCCCACTGA	Expression measurement
04582-L *BamH*I F	CATGGATCCATCCATGTTCCAATCGATC	Disruption of *MGG_04582*
04582-L *EcoR*I R	CATGAATTCTTGGCGCCGGTGTGTGGAT	Disruption of *MGG_04582*
04582-R *Xho*I F	CATCTCGAGGAAAAAAAGAAAGACAATA	Disruption of *MGG_04582*
04582-R *Kpn*I R	CATGGTACCCTCTAGCGTACACAGGAG	Disruption of *MGG_04582*
04582-CU	TGTGAATGACGACTTGTGCAAA	Confirmation of Δ*ebg1*
04582-CD	CAGGATGAAGGGCTTGAGC	Confirmation of Δ*ebg1*
HPT-U	GACAGACGTCGCGGTGAGTT	HPT primer
HPT-D	GTCCGAGGGCAAAGAAATAG	HPT primer
04582GTN *EcoR*I F	CATGAATTCTCCATGTTCCAATCGATCGA	Subcellular localization of EBG1
04582GTN *BamH*I R	CATGGATCCAGACGAGAAGAGGCGTCC	Subcellular localization of EBG1
3641-Pro-F	AAAGCTGGAGCTCCACCGCGGATGTATGTGGCTTTTA GTGGGACA	Subcellular localization of EF1α
3641-mCh-R	CGCCCTTGCTCACCATAAGCTTTTTCTTGGCGGCCTTG GCAGCGGA	Subcellular localization of EF1α
mCh-Hind-F	AAGCTTATGGTGAGCAAGGGCGAGGA	Subcellular localization of EF1α
mCh-Kpn-R	CTATAGGGCGAATTGGGTACCAGTGGAGATGTGGAGT GGGCGCTTA	Subcellular localization of EF1α
04582-6His RTN *EcoR*I F	CATGAATTCATGAAGGGCTTCACTGTCGC	Eukaryotic expression of EBG1
04582-6His RTN *BamH*I R	CATGGATCCTCAATGGTGATGGTGATGATG AGACGAGAAGAGGCGTCC	Eukaryotic expression of EBG1
YIP105-04582 F	AACCGCCAAAGGATCCATGAAGGGCTTCACT	Eukaryotic expression of EBG1 $^{E378Q\ E476Q}$
YIP105-04582 R	TAGAACTAGTGGATCCGTGGTGGTGGTGGTGGTGAG ACGAGAAGAGGCGT	Eukaryotic expression of EBG1 $^{E378Q\ E476Q}$
pIZGM-04582 F	AAACGGAAACCATGGAAGCTCACCGCCAGCACC	Pichia pastoris expression of EBG1
pIZGM-04582 R	GTACAGGTTTTCCTCGAGAGACGAGAAGAGGCGT	Pichia pastoris expression of EBG1
pIZGM-03641 F	TCGAAACGGAAACCATGGAAATGGGTAACAAGGAG	Pichia pastoris expression of EF1α
pIZGM-03641 R	GTACAGGTTTTCCTCGAGTTTCTTGGCGGCCT	Pichia pastoris expression of EF1α
pGEX-04582 F	GTATTTTCAGGGATCCATGAAGGGCTTCACT	Prokaryotic expression of

		EBG1
pGEX-04582 R	TGGTGGTGGTGCTCGAGAGACGAGAAGAGGCGT	Prokaryotic expression of EBG1
EBG1^{E378Q} *EcoR*I F1	CATGAATTCATGAAGGGCTTCACTGTC	Enzyme activity sites mutation
EBG1^{E378Q} R1	ACAAGGCCTGATTACCAACA ACACACATC	Enzyme activity sites mutation
EBG1^{E378Q} F2	GTAATCAGGCCTTGTTCAACGGCCACTGC	Enzyme activity sites mutation
EBG1^{E378Q} *BamH*I R2	CATGGATCCAGACGAGAAGAGGCGTC	Enzyme activity sites mutation
EBG1^{E476Q} *EcoR*I F1	CATGAATTCATGAAGGGCTTCACTGTC	Enzyme activity sites mutation
EBG1^{E476Q} R1	CCAGTCTGCATGATGTAGCCCTCCTTGCC	Enzyme activity sites mutation
EBG1^{E476Q} F2	CATCATGCAGACTGGCTGGCCCACCAATG	Enzyme activity sites mutation
EBG1^{E476Q} *BamH*I R2	CATGGATCCAGACGAGAAGAGGCGTCC	Enzyme activity sites mutation
04582 promoter GTN *Xho*I F	CATCTCGAGTCCATGTTCCAATCGATCG	Sites mutated complementation
04582 promoter GTN *Hind*III R	CATAAGCTTTGTTGCTGCTGCTGTTGC	Sites mutated complementation
EBG1$^{E378Q\ E476Q}$ native pro *Hind*III F	cgattggaacatggaaagcttATGAAGGGCTTCACTGTCGC	Sites mutated complementation
EBG1$^{E378Q\ E476Q}$ native pro *Hind*III R	gtcgacggtatcgataagcttAGACGAGAAGAGGCGTCCG	Sites mutated complementation
03641 promoter GTN *EcoR*I F	CGGAATTCTGGCTCTGGATACTGAAGGC	Overexpression
03641 promoter GTN *BamH*I R	CGGGATCCTTTGGCGGTTTGGTGCTC	Overexpression
OE EBG1$^{E378Q\ E476Q}$ GFP *BamH*I F	caccaaaccgccaaaggatccATGAAGGGCTTCACTGTCGC	Overexpression of EBG1$^{E378Q\ E476Q}$
OE EBG1$^{E378Q\ E476Q}$ GFP *BamH*I R	acctctagaactagtggatccAGACGAGAAGAGGCGTCCG	Overexpression of EBG1$^{E378Q\ E476Q}$
OE SLP1-GFP F	caccaaaccgccaaaggatccATGCAGTTCGCTACCATCACC	Co-immunoprecipitation (Control)
OE SLP1-GFP R	acctctagaactagtggatccGTTCTTGCAGATGGGGATGTTG	Co-immunoprecipitation (Control)
03641-3Flag TH *EcoR*I F	CATGAATTCAGTAAGCTGCCAAGAATCCA	Co-immunoprecipitation
03641-3Flag TH *BamH*I R	CATGGATCCTTTCTTGGCGGCCTTGGC	Co-immunoprecipitation
03641-L *BamH*I F	CATGGATCCCTGTGGCCAACGTGAAT	Disruption of *MGG_03641*
03641-L *EcoR*I R	CATGAATTCAGCAGCCCATATGCAAGA	Disruption of *MGG_03641*
03641-R *Cla*I F	CATATCGATAGGACATTCGCCGTGGTA	Disruption of *MGG_03641*
03641-R *Xho*I R	CATCTCGAGTTCCTCCTGTCTTTTAAACA	Disruption of *MGG_03641*
03641-CU	CATAGTAACTAGATCATGGTAA	Confirmation of Δ*ef1*α
03641-CD	TGTGTCGCCTTTGTGCTG	Confirmation of Δ*ef1*α
03641GTN *EcoR*I F	CGGAATTCTGGCTCTGGATACTGAAGGC	Complementation of EF1α
03641GTN *BamH*I R	CATGGATCCTTTCTTGGCGGCCTTGGC	Complementation of EF1α
EBG1-ΔSP BD *EcoR*I F	CATGAATTCATG CGCCAGCACCGCCAGG	Yeast two-hybrid
EBG1-ΔSP BD *BamH*I R	CATGGATCCTCAAGACGAGAAGAGGCGTC	Yeast two-hybrid
EBG1-N BD *EcoR*I F	CATGAATTCATGCGCCAGCACCGCCAGG	Yeast two-hybrid
EBG1-N BD *BamH*I R	CATGGATCCTCAGGTAGCAGGGGGCGAAGA	Yeast two-hybrid
EBG1-C BD *EcoR*I F	CATGAATTCATGTCGCCCCCTAAGCTGGG	Yeast two-hybrid

EBG1-C BD *BamH*I R	CATGGATCCTCAAGACGAGAAGAGGCGTC	Yeast two-hybrid
EF1α AD *EcoR*I F	CATGAATTCATGGGTAACAAGGAGAAGA	Yeast two-hybrid
EF1α AD *BamH*I R	CATGGATCCTTATTTCTTGGCGGCCTTG	Yeast two-hybrid
EF1α-D1 AD *EcoR*I F	ATGAATTCATGAAGAGCCATCTTAACGTCGT	Yeast two-hybrid
EF1α-D1 AD *BamH*I R	CATGGATCCTTAGATCATGTGGTCACCGTTG	Yeast two-hybrid
EF1α-D2 AD *EcoR*I F	CATGAATTCATGGGCACAGTCCCCGTCG	Yeast two-hybrid
EF1α-D2 AD *BamH*I R	CATGGATCCTAAGACGTTACCACGGCGAAT	Yeast two-hybrid
EF1α-D3 AD *EcoR*I F	CATGAATTCATGGGCTGCGCTTCGTTCAAC	Yeast two-hybrid
EF1α-D3 AD *BamH*I R	CATGGATCCTAAGACGCTCTTGATGACACC	Yeast two-hybrid
FgEBG1-ΔSP BD F	ATGGCCATGGAGGCCGAATTCATGTACCCTCACCACC GACGTG	Yeast two-hybrid
FgEBG1-ΔSP BD R	TCGACGGATCCCCGGGAATTCTTAGTAGCCGCCGATG AA	Yeast two-hybrid
FgEF1 α-ΔSP AD F	GCCATGGAGGCCAGTGAATTCATGGGTAAGGAGGAG AAG	Yeast two-hybrid
FgEF1 α-ΔSP AD F	CAGCTCGAGCTCGATGGATTTATTTCTTGGCAGCCTT GGC	Yeast two-hybrid
08162 AD *EcoR*I F	CATGAATTCATGTCTGCTACTTTCAG	Yeast two-hybrid
08162 AD *Pst*I R	CATCTGCAGTTATTTGAGGATGCGAGT	Yeast two-hybrid
02504 AD *EcoR*I F	CATGAATTCATGTCTCGACACAGTGC	Yeast two-hybrid
02504 AD *BamH*I R	CATGGATCCCTATTCGATAATTCCAGC	Yeast two-hybrid
pGWB414:EBG1:3HA F	CACCATGAAGGGCTTCACTGTCGC	*A. tumefaciens* infiltration
pGWB414:EBG1:3HA R	AGACGAGAAGAGGCGTC	*A. tumefaciens* infiltration
pGWB414:EBG1-N:3HA F	CACCATGAAGGGCTTCACTGTCGC	*A. tumefaciens* infiltration
pGWB414:EBG1-N:3HA R	GGTAGCAGGGGGCGAAGA	*A. tumefaciens* infiltration
pGWB414:EBG1-C:3HA F	CACCATGAAGGGCTTCACTGTCGCCACTGC CGCTGTCGCGGCCCTCTCCGGAGGTGTCAG CGCTCACTCGCCCCCTAAGCTGGG	*A. tumefaciens* infiltration
pGWB414:EBG1-C:3HA R	AGACGAGAAGAGGCGTC	*A. tumefaciens* infiltration
pGWB414:EF1α:3HA F	CACCATGGGTAACAAGGAGAAGA	*A. tumefaciens* infiltration
pGWB414:EF1α:3HA R	TTTCTTGGCGGCCTTG	*A. tumefaciens* infiltration
pGWB414:INF1:3HA F	CACCATGAACTTTCGTGCTCTGT	*A. tumefaciens* infiltration
pGWB414:INF1:3HA R	CGACGCACACGTAGACGAG	*A. tumefaciens* infiltration
pGWB414:GFP:3HA F	CACCATGGTGAGCAAGGGCGAGG	*A. tumefaciens* infiltration
pGWB414:GFP:3HA R	AAGATCTACCATGTACAG	*A. tumefaciens* infiltration
pJL12:EBG1-ΔSP-3FLAG F	TCTGATCAAGAGACAGGATCCATGCGCCAGCACCGCC AGGCT	*A. tumefaciens* infiltration
pJL12:EBG1-ΔSP-3FLAG R	GTAGTCAGCGGCCGCTCTAGAAGACGAGAAG AGGCGTCC	*A. tumefaciens* infiltration
pJL12:FgEBG1-3FLAG F	TCTGATCAAGAGACAGGATCCATGAAGGGAGGAATTG CC	*A. tumefaciens* infiltration
pJL12:FgEBG1-3FLAG R	GTAGTCAGCGGCCGCTCTAGGTAGCCGCCGATGAAG GC	*A. tumefaciens* infiltration
pJL12:EF1α-HA F	TCTGATCAAGAGACAGGATCCATGGGTAACAAGGAGA	*A. tumefaciens* infiltration

	AG	
pJL12:EF1α-HA R	TGGGTAAGCGGCCGCTCTAGATTTCTTGGCGGCCTTGGC	*A. tumefaciens* infiltration
pJL12: FgEF1α-HA F	TCTGATCAAGAGACAGGATCCATGGGTAAGGAGGAGAAG	*A. tumefaciens* infiltration
pJL12: FgEF1α-HA R	TGGGTAAGCGGCCGCTCTAGATTTCTTGGCAGCCTTGGC	*A. tumefaciens* infiltration
35S::EBG1-Myc-nYFP F	ATTAACAAGGCCATTACGGCCATGAAGGGCTTCACTGTC	BiFC assay
35S::EBG1-Myc-nYFP R	AACTGATTGGCCGAGGCGGCCCCGAGACGAGAAGAGGCGTC	BiFC assay
35S::HA-cYFP-EF1α F	ATTAACAAGGCCATTACGGCCATGGGTAACAAGGAGAAGA	BiFC assay
35S::HA-cYFP-EF1α R	AACTGATTGGCCGAGGCGGCCCCGTTTCTTGGCGGCCTTG	BiFC assay
NbBAK1 qPCR F	GCAGAGTACGCCTACGGAAC	Expression measurement
NbBAK1 qPCR R	TCCGGTCGGATATTTGAAGTGG	Expression measurement
NbEF1α qPCR F	AAGGTCACCAAGGCTGCTCA	Expression measurement
NbEF1α qPCR R	GGGAGCGGATACCAGTCATAC	Expression measurement
pSUC2 EBG1 F	TTTAATTAAGAATTCATGAAGGGCTTCACTGTCGC	Secretion identification of EBG1
pSUC2 EBG1 R	AGGGAGAACCTCGAGAGACGAGAAGAGGCGTC	Secretion identification of EBG1
pSUC2 EBG1-ΔSP F	TTTAATTAAGAATTCATGCGCCAGCACCGCCAGG	Secretion identification of EBG1-ΔSP
pSUC2 EBG1-ΔSP R	AGGGAGAACCTCGAGAGACGAGAAGAGGCGTCC	Secretion identification of EBG1-ΔSP
pSUC2-F	CAGATGTCGTTGTTCCAGAGC	Sequencing Primer
pSUC2-R	TCATCGTACCACAACCCATTTG	Sequencing Primer
T7	TAATACGACTCACTATAGGG	Sequencing Primer
