# Supplementary material for: Plant immunity suppression by an exo-β-1,3-glucanase and an elongation factor 1α of the rice blast fungus
Source: Nat Commun. 2023 Sep 7;14:5491. doi: 10.1038/s41467-023-41175-z (PMC10484928; doi:10.1038/s41467-023-41175-z)
Supplement: Supplementary file 2 — Description of Supplementary Data [file 41467_2023_41175_MOESM2_ESM.pdf]

Description of Additional Supplementary Files:

Supplementary Data 1: Identification of peptides of MoEf1α and MoEbg1 in wild-type P131 liquid culture medium by LC-MS/MS. Secreted proteins of wild-types P131 were extracted from liquid medium.

Supplementary Data 2: LC-MS/MS Identified MoEf1α peptides from barley apoplastic fluid after plants infected with P131.
